# Supplementary material for: Associations between picocyanobacterial ecotypes and cyanophage host genes across ocean basins and depth
Source: PeerJ. 2023 Feb 28;11:e14924. doi: 10.7717/peerj.14924 (PMC9983427; doi:10.7717/peerj.14924)
Supplement: Supplemental Information 1 — Data from stations not highlighted in the article. [file peerj-11-14924-s001.pdf]

Supplemental Figures for “Associations between picocyanobacterial ecotypes and cyanophage host genes across ocean basins and depth” by Fuchsman, Garcia-Prieto, Hays, and Cram.

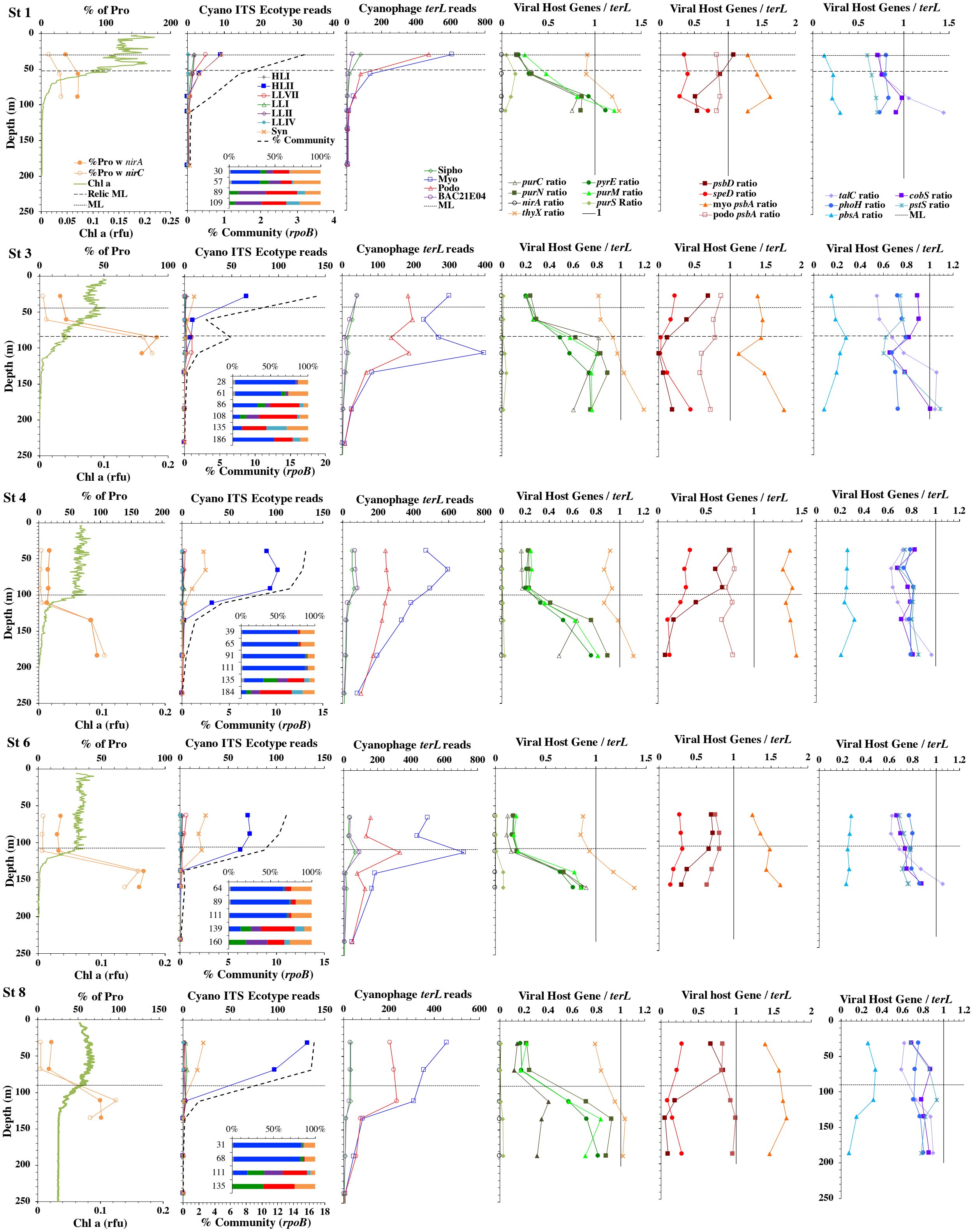

Figure S1. Stations from GA03 section 1, a North Atlantic shelf to slope transect. Column A) The % of *Prochlorococcus* with nitrite assimilation genes *nirA* and *nirC*, and chlorophyll. Column B) Cyanobacterial internal transcribed spacer (ITS) ecotype normalized reads and percent of prokaryote community (RNA polymerase, *rpoB*; dashed bold line). Insert: Cyanobacterial ecotypes as % of Picocyanobacteria. Colors match the symbols. Ecotypes are designated by gray (HLI), blue (HLII), green (LLI), purple (LLII), red (LLVII or NC1), light blue (LLIV) and orange (*Synechococcus*). Column C) Cyanophage abundance as determined by *terL* normalized reads. Column D-F) The proportion of cyanophage with a host gene as measured by host gene/*terL* ratio. Full gene names and function can be found in Table 1. The mixed layer (ML) is indicated by the dotted line.

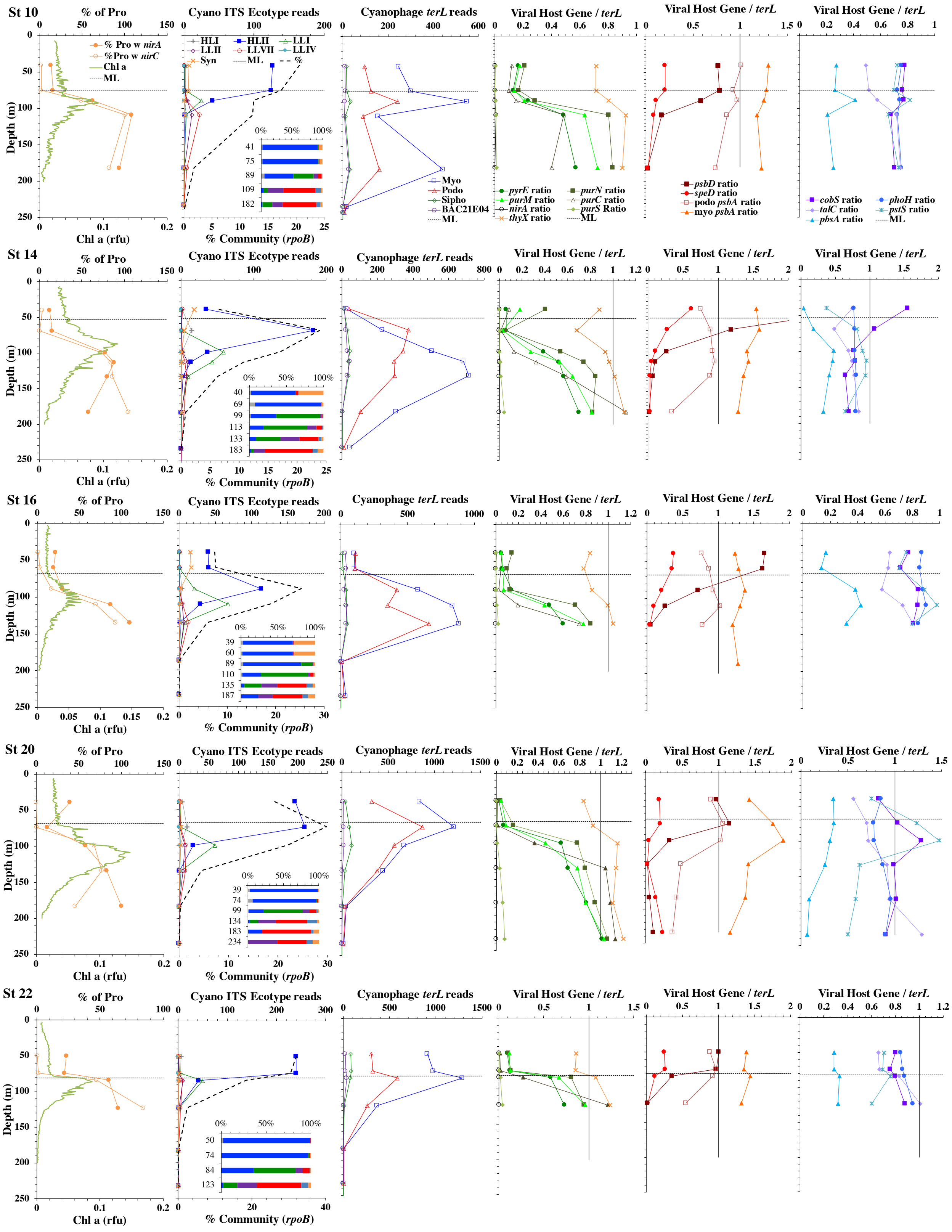

Figure S2. Data from GA03 section 2 crossing the North Atlantic subtropical gyre. Panels are the same as Figure S1.

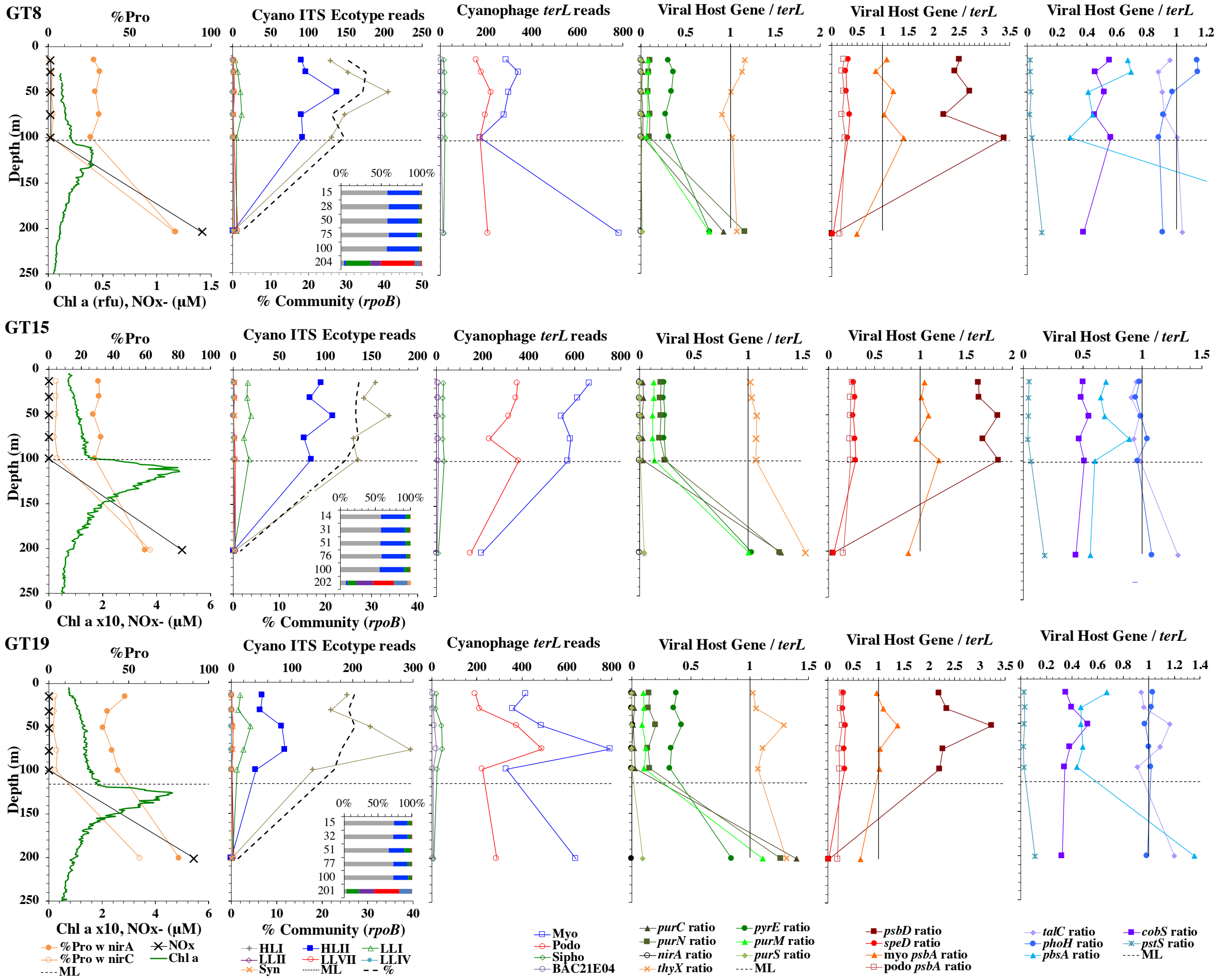

Figure S3. Stations from South Pacific transect GP13. Column A) The % of *Prochlorococcus* with nitrite assimilation genes *nirA* and *nirC*, NO_x^- concentrations, and chlorophyll multiplied by 10. Column B) Cyanobacterial ITS ecotype normalized reads and percent of prokaryote community (*rpoB*; dashed bold line). Insert: Cyanobacterial ecotypes as % of Picocyanobacteria. Colors match the symbols. Column C) Cyanophage abundance as determined by *terL* normalized reads. Column D-F) The proportion of cyanophage with a host gene as measured by host gene/*terL* ratio. The mixed layer (ML) is indicated by the dotted line. $NO_x^- = NO_3^- + NO_2^-$.

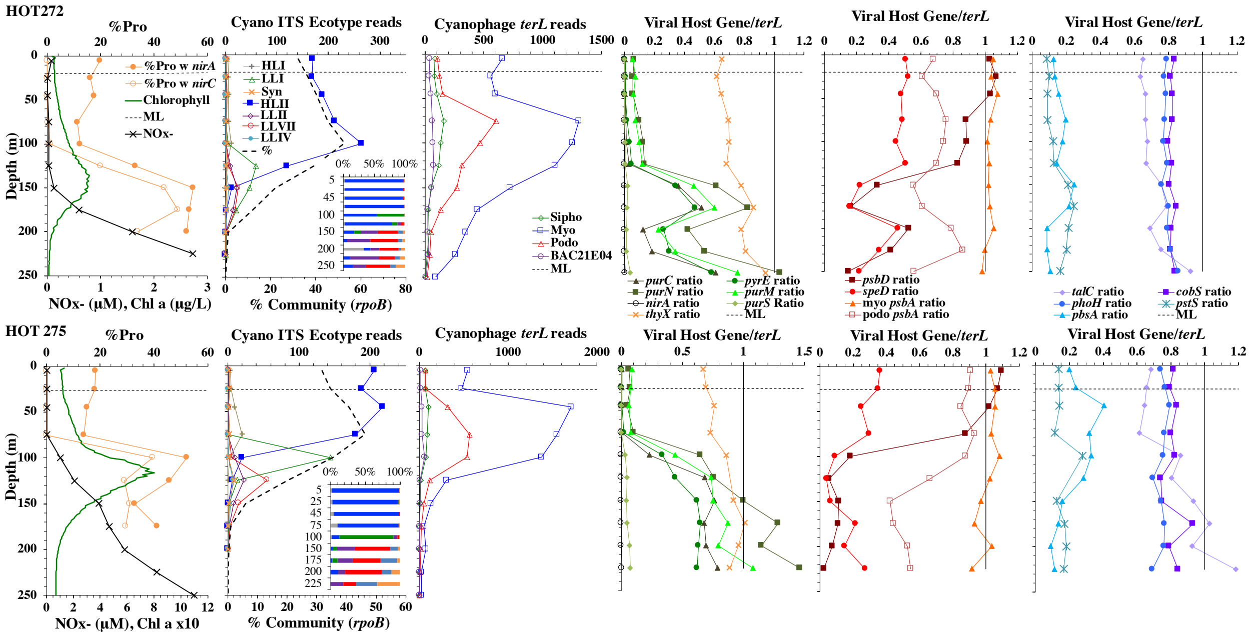

Figure S4 Dataset from HOT 2015 (HOT 272 [May] top, HOT 275 [Aug] bottom). Panels are the same as for Figure S3.

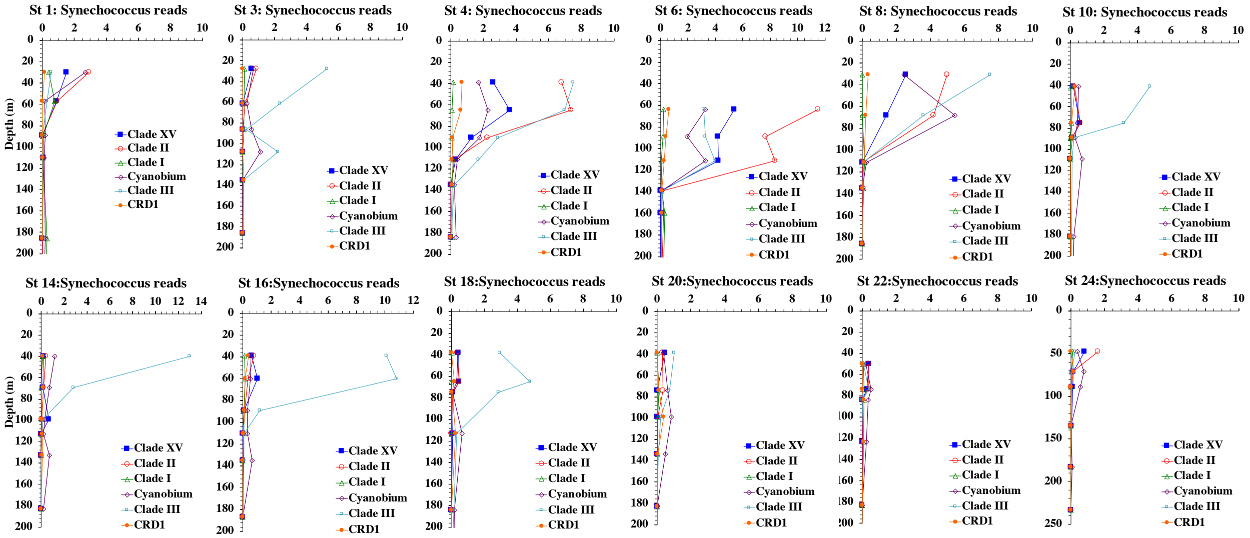

Figure S5. *Synechococcus* ecotypes on North Atlantic transect GA03. Clades were based on Lavin et al (2010).

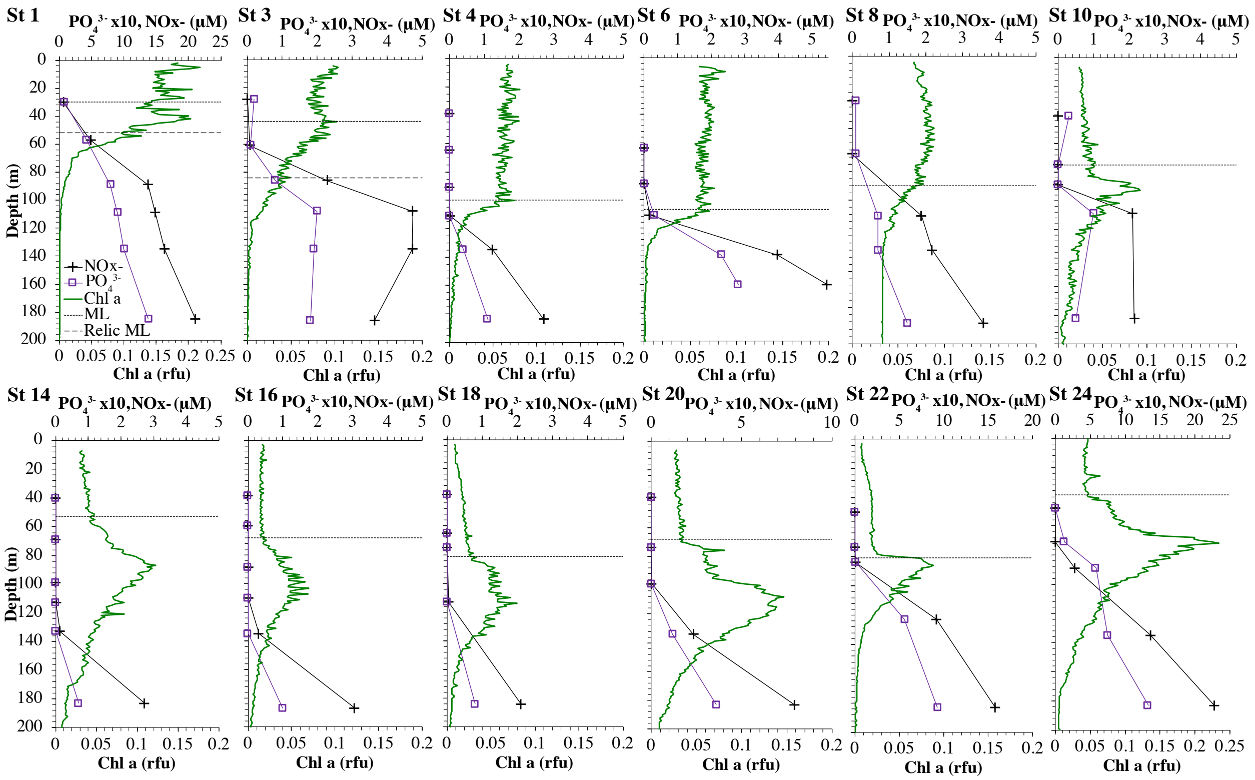

Figure S6. NO_x^- and PO_4^{3-} concentrations and chlorophyll fluorescence for the GA03 transect. $NO_x^- = NO_3^- + NO_2^-$. Mixed layer is the dotted line. Relic mixed layer is the dashed line.

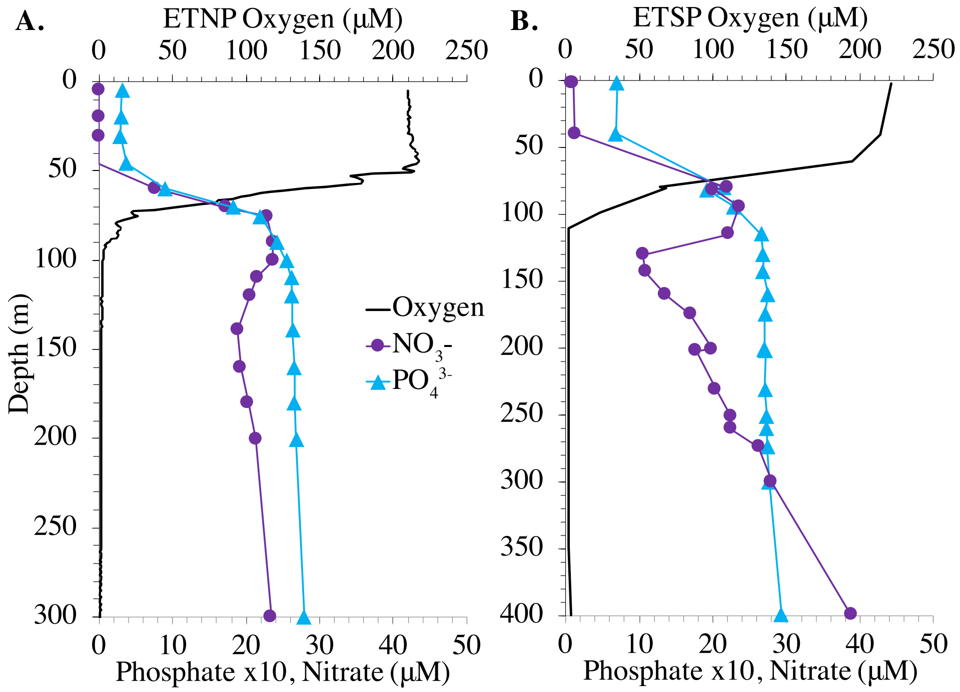

Figure S7. Oxygen, nitrate and phosphate depth profiles for the ETNP St 136 and ETSP St 9. Phosphate concentrations were multiplied by 10 to fit on the axis.

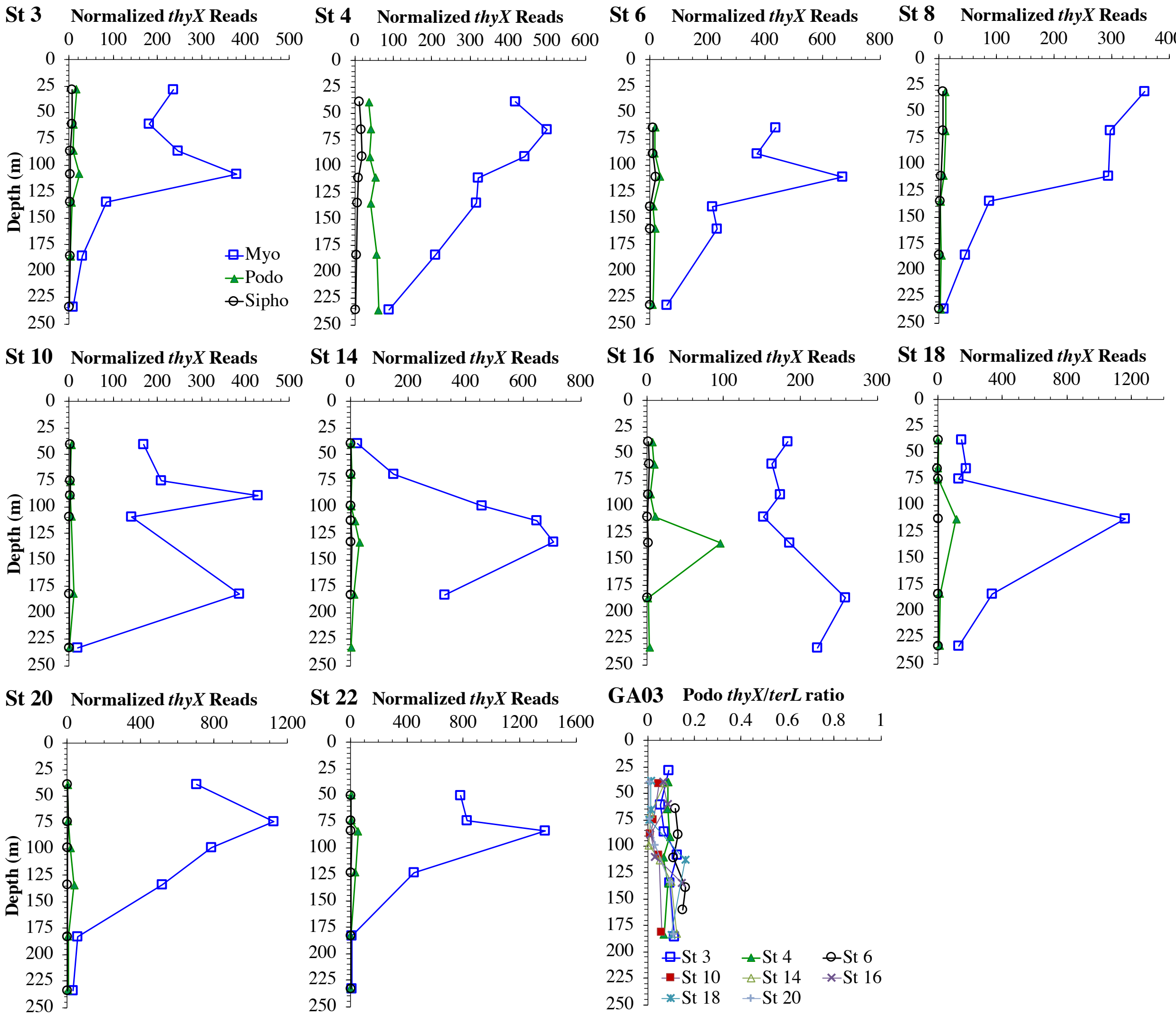

Figure S8. A comparison of normalized reads for *thyX* types at stations in North Atlantic transect GA03, and podo *thyX/terL* ratios for these stations.

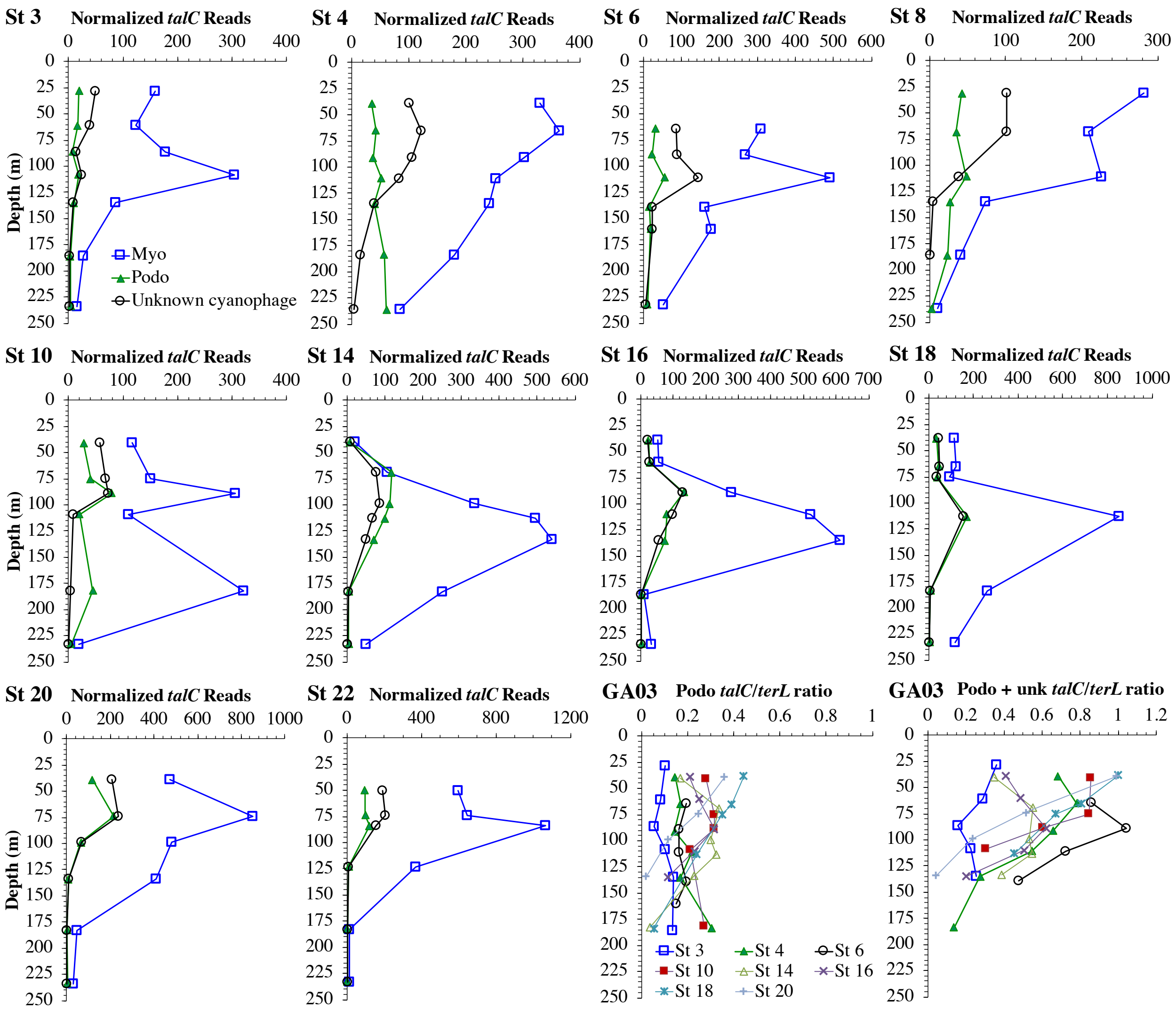

Figure S9. A comparison of normalized reads for *talC* types at stations in North Atlantic transect GA03, and podo *talC/terL* ratios for these stations. The final panel represents the theoretical situation were the unknown cyanophage contigs are from podo-cyanophage.

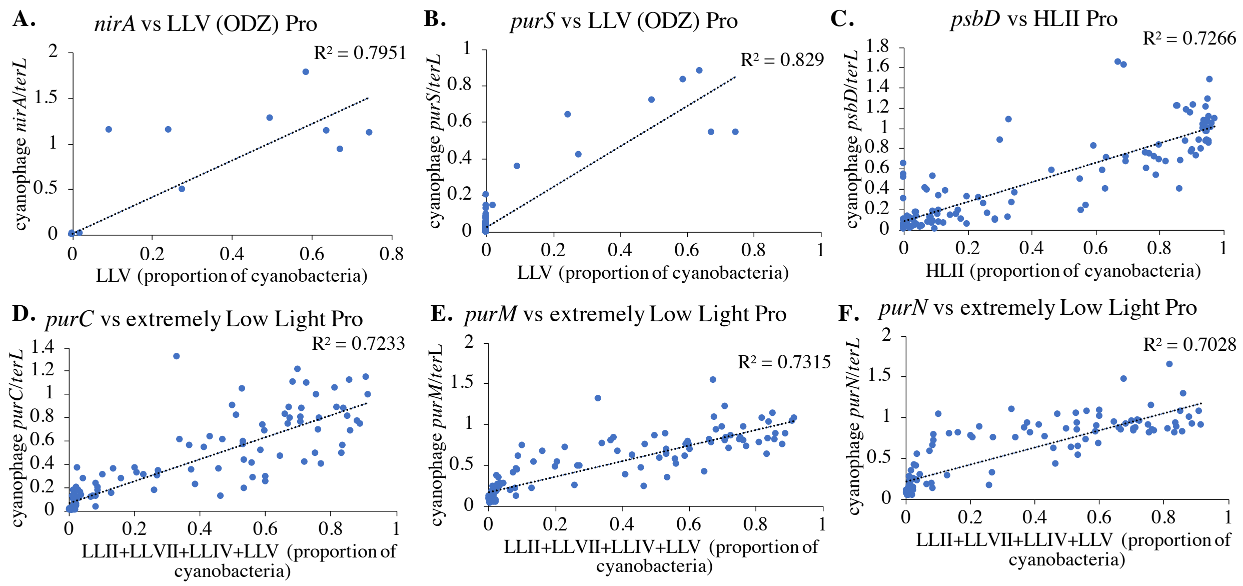

Figure S10. Linear correlations between ecotypes and viral host gene/*terL* ratios.

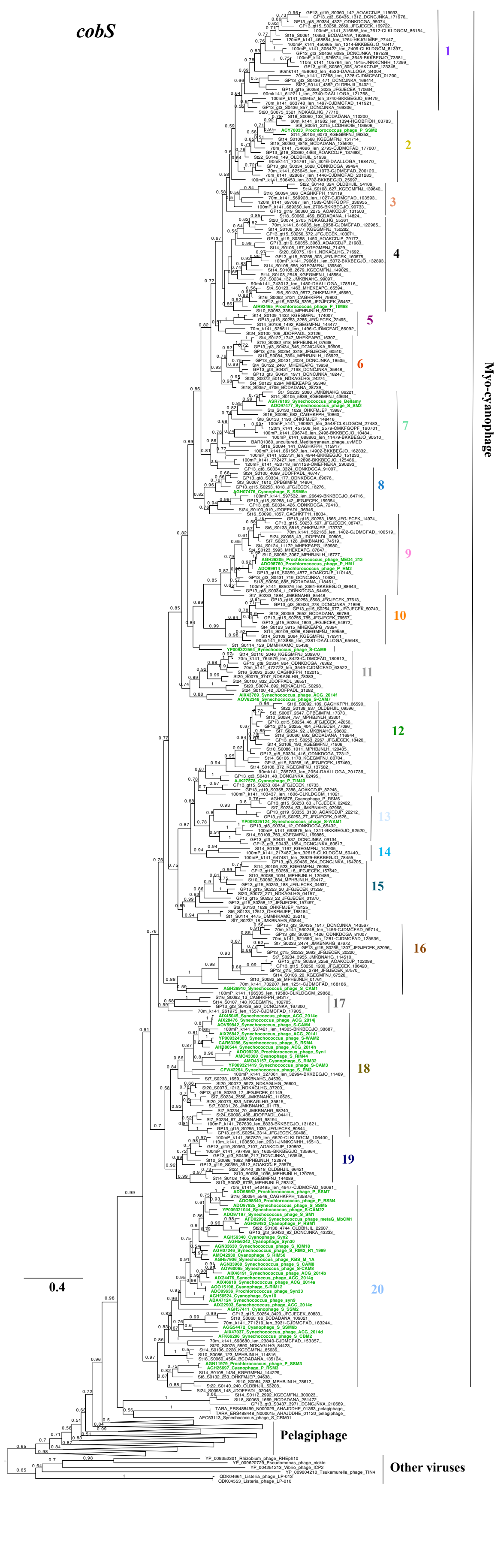

cobS
1
2
3
4
5
6
7
8
9
10
11
12
13
14
15
16
17
18
19
20
Myo-cyanophage
Pelagiphage
Other viruses
0.4

Figure S11. Amino acid phylogenetic tree for *cobS* with phylotypes marked. Colors match colors in *cobS* depth profiles. Bootstraps are indicated on the nodes. Green names indicate cyanophage isolates.

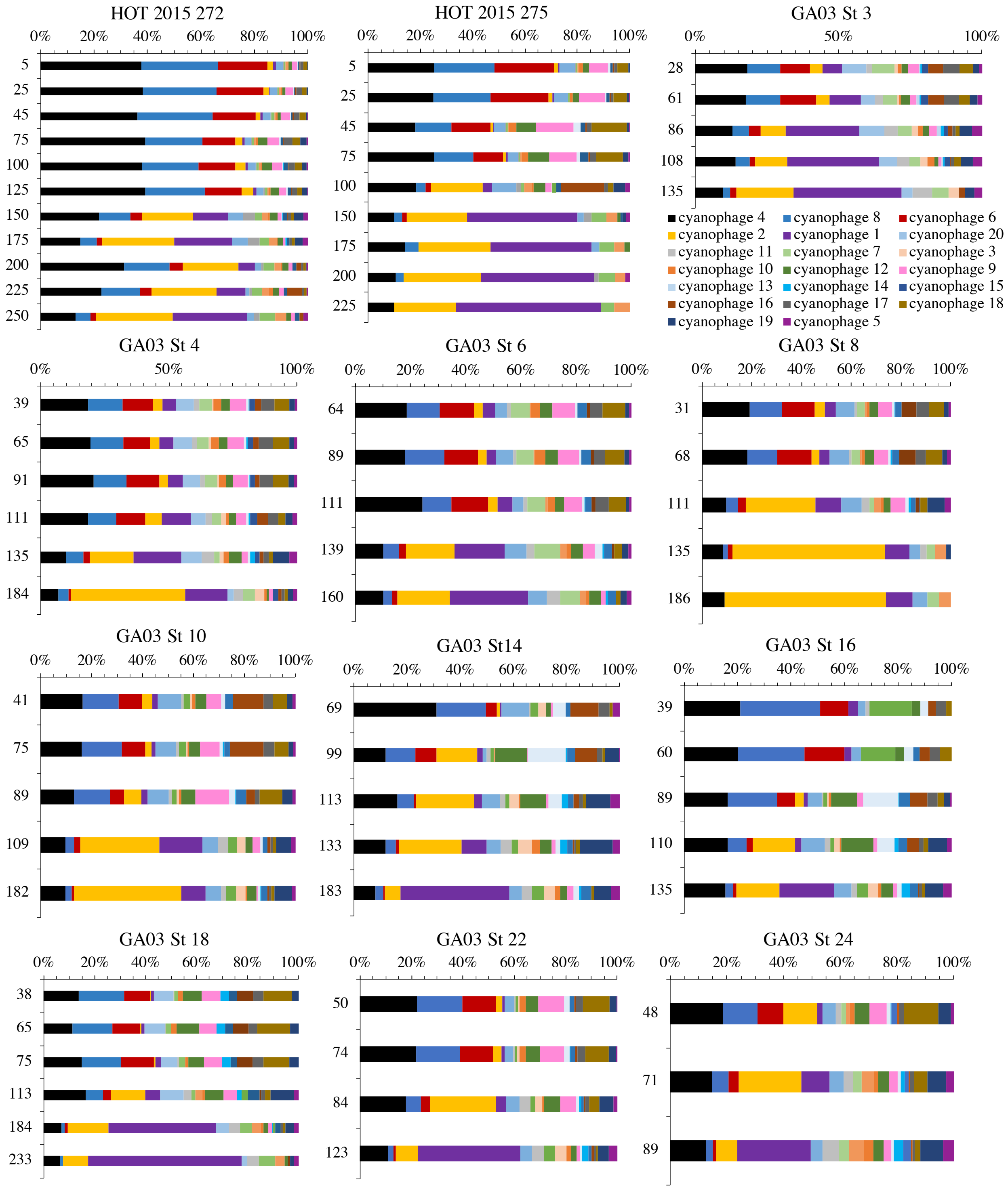

Figure S12. *cobS* phylotypes across depth at oxic stations. Phylotypes are ordered by abundance. The phylogenetic tree with phylotypes marked is seen in Figure S11.

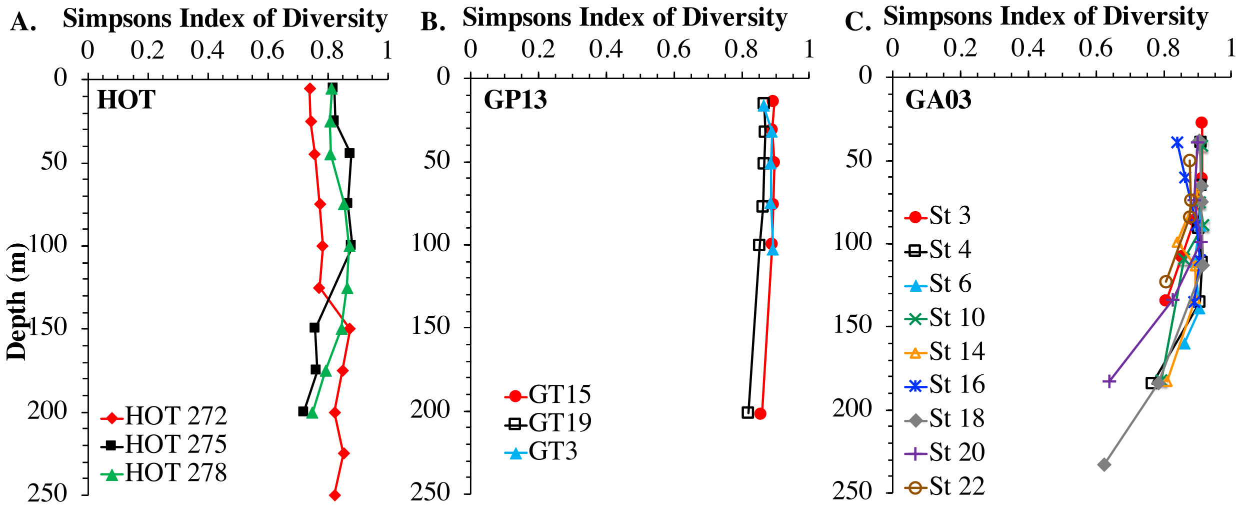

Figure S13. Simpson's Diversity Index (1-D) of *cobS* phylotypes for myo-cyanophage. A) North Pacific HOT samples from 2015 for HOT 272 (May), HOT 275 (Aug), HOT 278 (Nov), B) South Pacific GP13 stations, and C) North Atlantic GA03 transect.

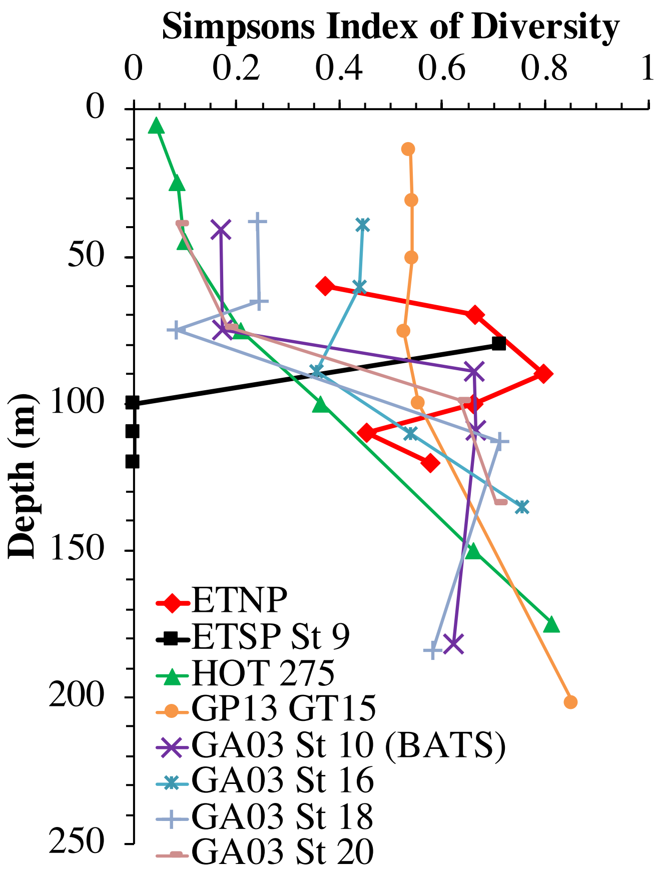

Figure S14. Simpson's Diversity Index (1-D) of ITS cyanobacterial ecotypes for select stations. The ETNP ODZ samples are 110 m and 120 m, and the ETSP ODZ samples are 100-120 m.
