# Supplementary material for: Associations between picocyanobacterial ecotypes and cyanophage host genes across ocean basins and depth
Source: PeerJ. 2023 Feb 28;11:e14924. doi: 10.7717/peerj.14924 (PMC9983427; doi:10.7717/peerj.14924)
Supplement: Supplemental Information 2 — Phylogenetic trees for cobS, nirA, nirC, pstS, speD, pbsA, psbA, psbD, talC, thyX, pyrE, purC, purN, purM, phoH. For each tree, bootstraps can be found over the nodes. Bootstraps are between 0-1 and those below 0.50 are not shown. References shown in green are isolates, and sequences shown in blue have been verified to be myo-cyanophage. Most blue sequences were verified in Fuchsman et al. (2021). [file peerj-11-14924-s002.pdf]

Supplemental Data for
Associations between picocyanobacterial ecotypes and cyanophage host genes across ocean basins

Clara A. Fuchsman, David Garcia-Prieto, Matthew D. Hays, Jacob A. Cram

Supplemental Data contains the phylogenetic trees created in this paper. For each tree, bootstraps can be found over the nodes. Bootstraps are between 0-1 and those below 0.50 are not shown. References shown in green are isolates, and sequences shown in blue have been verified to be myo-cyanophage. Most blue sequences were verified in Fuchsman et al (2021).

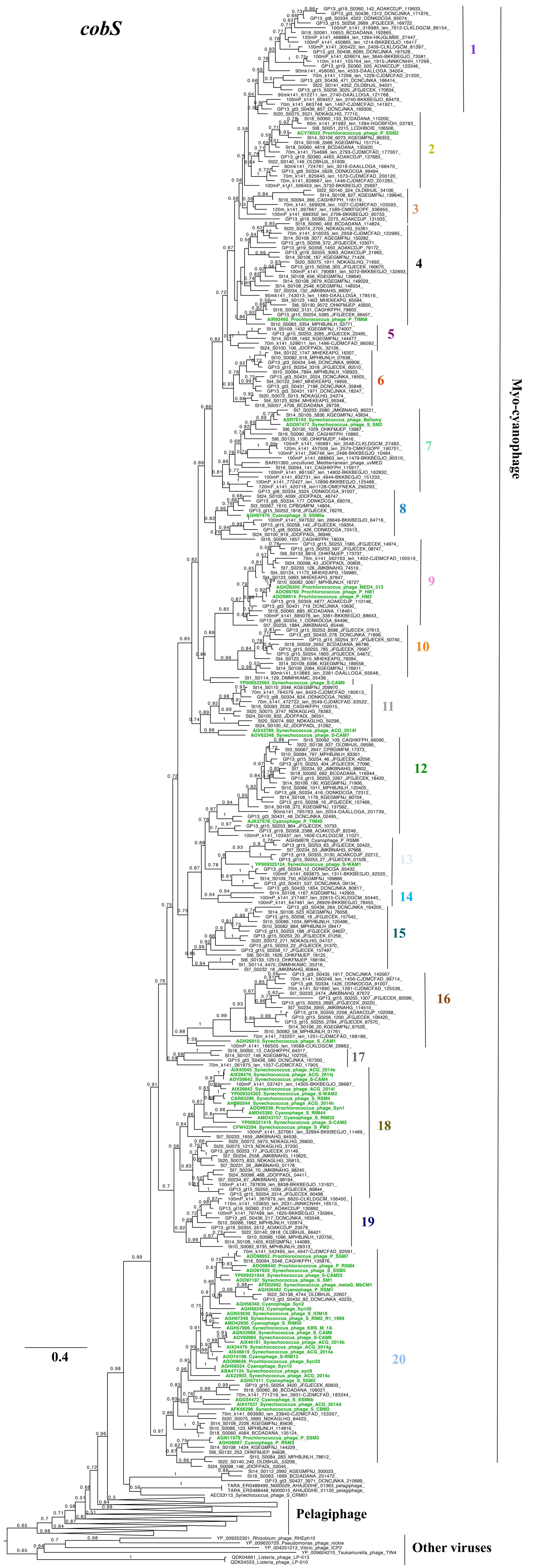

cobS
1
2
3
4
5
6
7
8
9
10
11
12
13
14
15
16
17
18
19
20
Myo-cyanophage
Pelagiphage
Other viruses
0.4

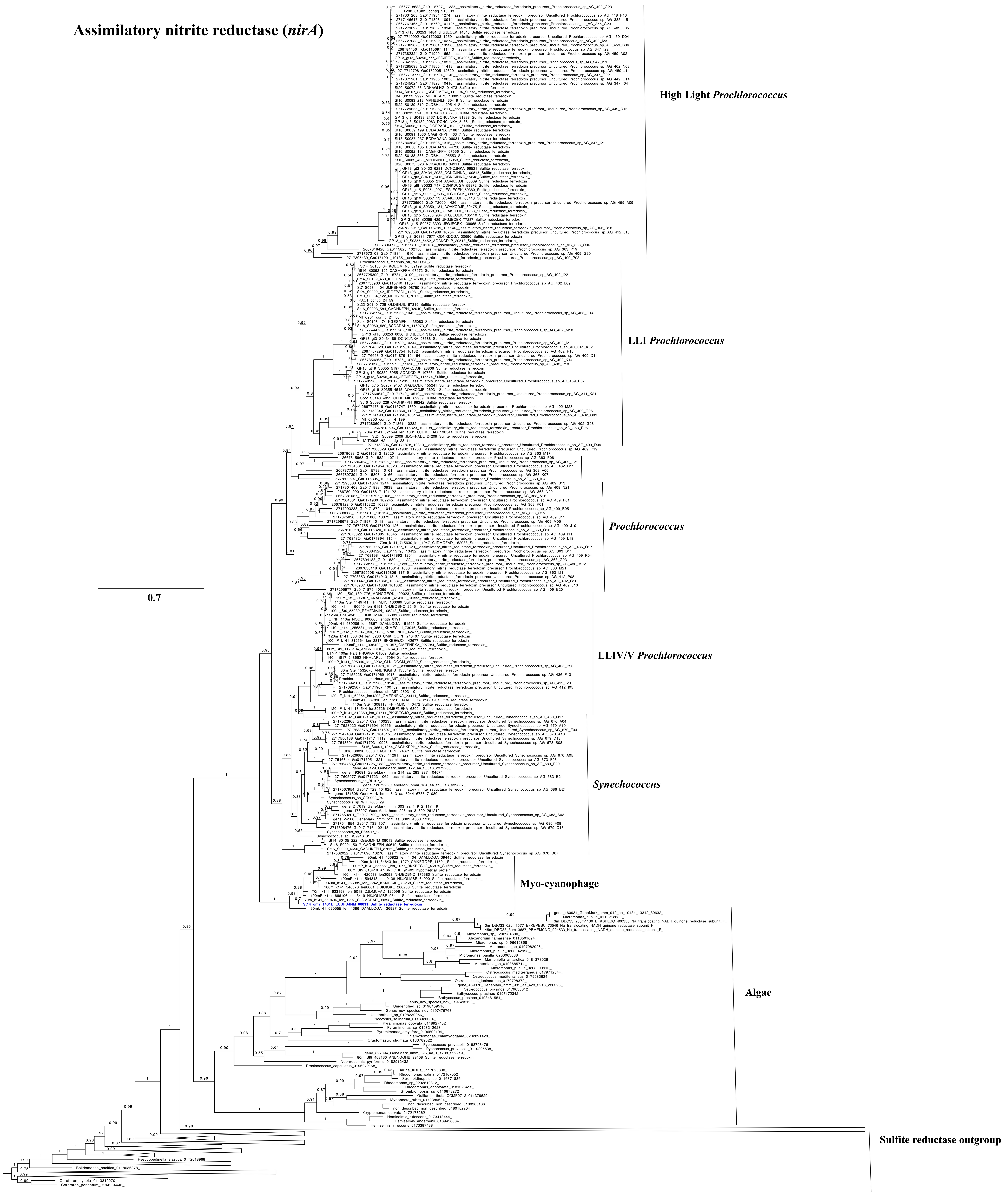
Assimilatory nitrite reductase (nirA)
High Light Prochlorococcus
LLI Prochlorococcus
Prochlorococcus
LLIV/V Prochlorococcus
Synechococcus
Myo-cyanophage
Algae
Sulfite reductase outgroup
0.7

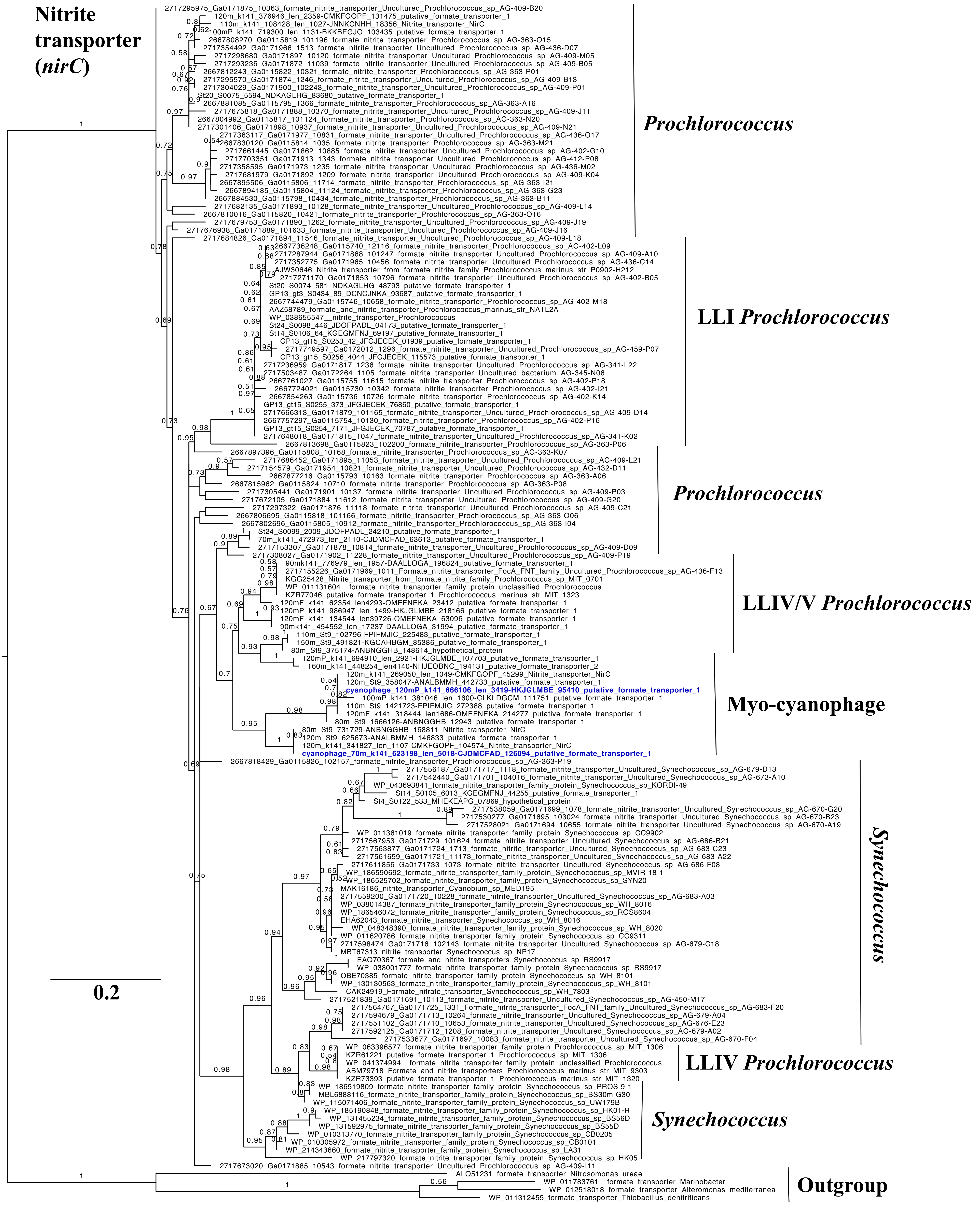
Nitrite transporter (*nirC*)
Prochlorococcus
LLI *Prochlorococcus*
Prochlorococcus
LLIV/V *Prochlorococcus*
Myo-cyanophage
Synechococcus
LLIV *Prochlorococcus*
Synechococcus
Outgroup
0.2

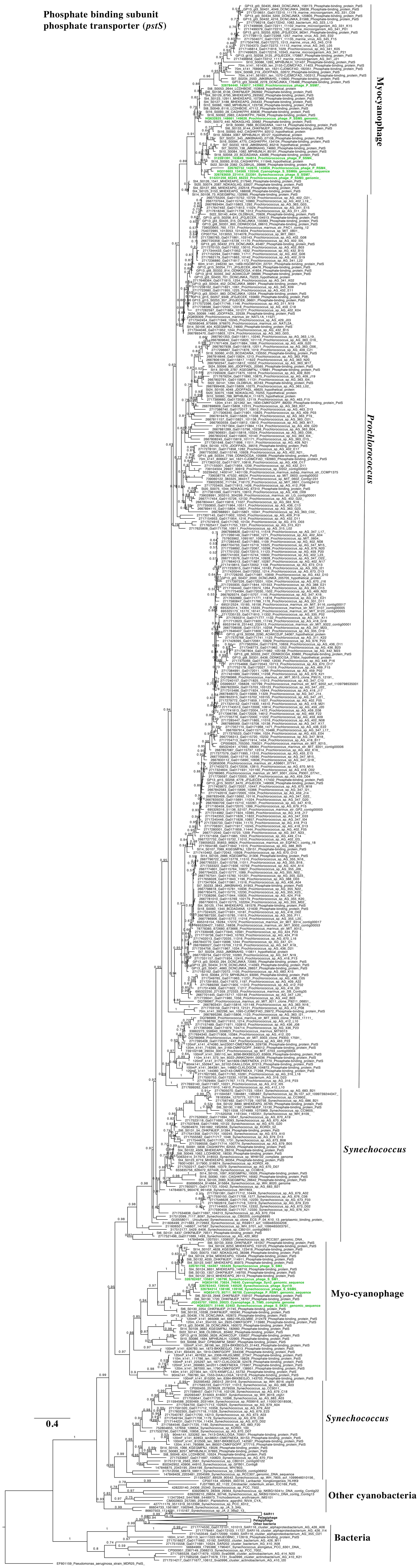
Phosphate binding subunit phosphate transporter (*pstS*)
Myocyanophage
Prochlorococcus
Synechococcus
Myo-cyanophage
Synechococcus
0.4
Other cyanobacteria
Bacteria

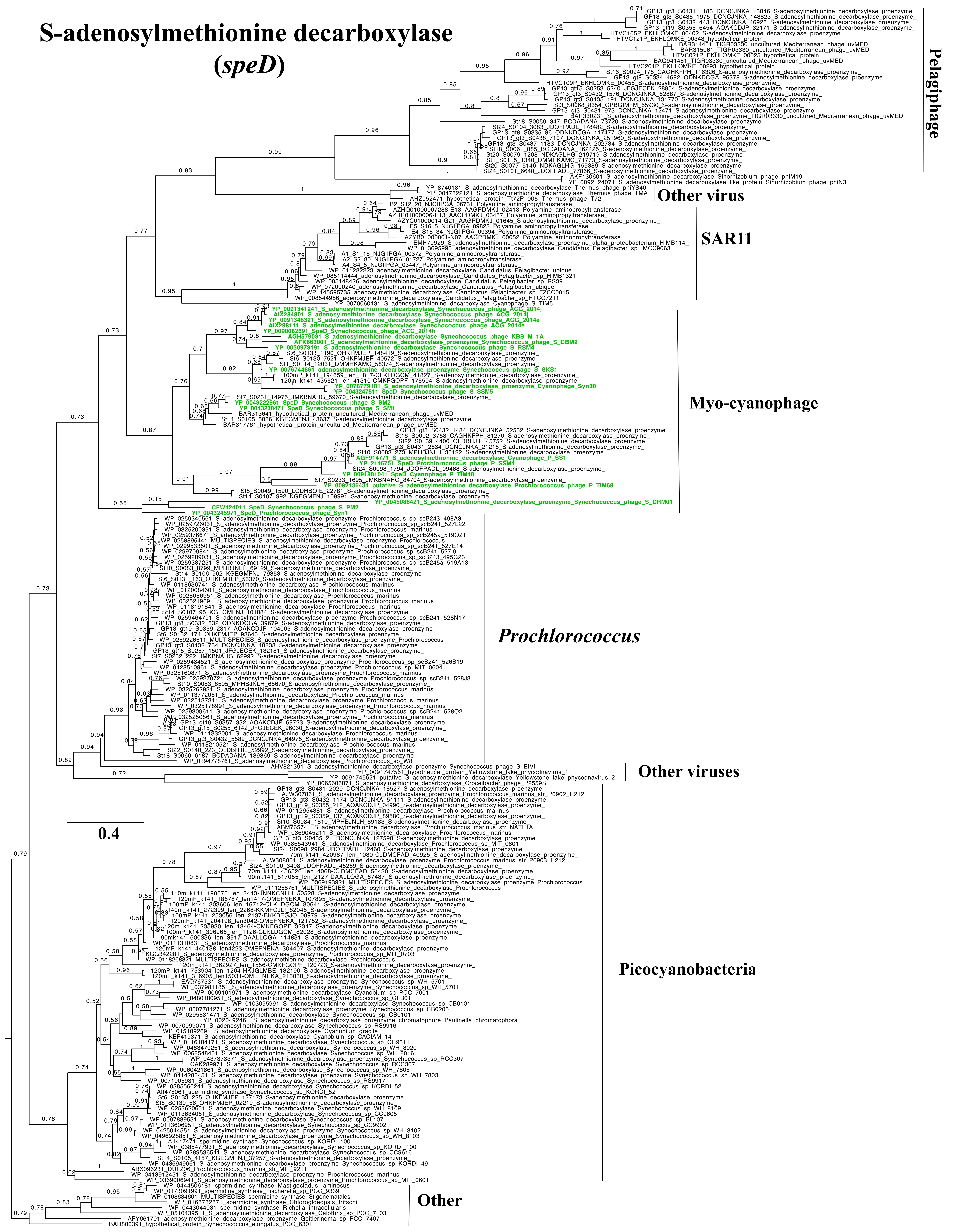
S-adenosylmethionine decarboxylase
(speD)
Pelagiphage
Other virus
SAR11
Myo-cyanophage
Prochlorococcus
Other viruses
0.4
Picocyanobacteria
Other

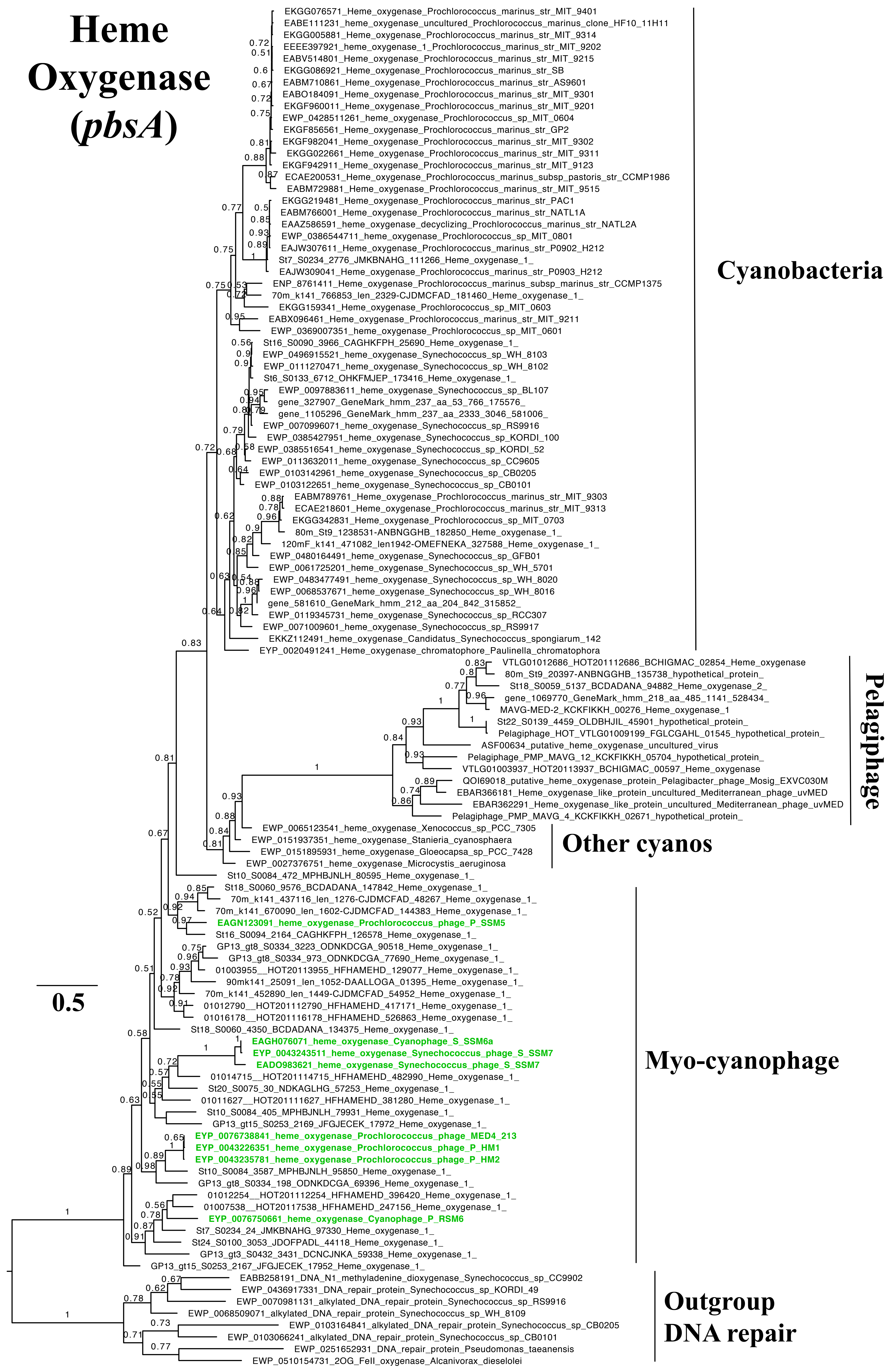

Heme Oxygenase (pbsA)
Cyanobacteria
Pelagiphage
Other cyanos
Myo-cyanophage
Outgroup DNA repair
0.5
EKGG076571_Heme_oxygenase_Prochlorococcus_marinus_str_MIT_9401
EABE111231_heme_oxygenase_uncultured_Prochlorococcus_marinus_clone_HF10_11H11
EKGG005881_Heme_oxygenase_Prochlorococcus_marinus_str_MIT_9314
EEEE397921_heme_oxygenase_1_Prochlorococcus_marinus_str_MIT_9202
EABV514801_Heme_oxygenase_Prochlorococcus_marinus_str_MIT_9215
EKGG086921_Heme_oxygenase_Prochlorococcus_marinus_str_SB
EABM710861_Heme_oxygenase_Prochlorococcus_marinus_str_AS9601
EABO184091_Heme_oxygenase_Prochlorococcus_marinus_str_MIT_9301
EKGF960011_Heme_oxygenase_Prochlorococcus_marinus_str_MIT_9201
EWP_0428511261_heme_oxygenase_Prochlorococcus_sp_MIT_0604
EKGF856561_Heme_oxygenase_Prochlorococcus_marinus_str_GP2
EKGF982041_Heme_oxygenase_Prochlorococcus_marinus_str_MIT_9302
EKGG022661_Heme_oxygenase_Prochlorococcus_marinus_str_MIT_9311
EKGF942911_Heme_oxygenase_Prochlorococcus_marinus_str_MIT_9123
ECAE200531_Heme_oxygenase_Prochlorococcus_marinus_subsp_pastoris_str_CCMP1986
EABM729881_Heme_oxygenase_Prochlorococcus_marinus_str_MIT_9515
EKGG219481_Heme_oxygenase_Prochlorococcus_marinus_str_PAC1
EABM766001_Heme_oxygenase_Prochlorococcus_marinus_str_NATL1A
EAAZ586591_heme_oxygenase_decyclizing_Prochlorococcus_marinus_str_NATL2A
EWP_0386544711_heme_oxygenase_Prochlorococcus_sp_MIT_0801
EAJW307611_Heme_oxygenase_Prochlorococcus_marinus_str_P0902_H212
St7_S0234_2776_JMKBNAHG_111266_Heme_oxygenase_1_
EAJW309041_Heme_oxygenase_Prochlorococcus_marinus_str_P0903_H212
ENP_8761411_Heme_oxygenase_Prochlorococcus_marinus_subsp_marinus_str_CCMP1375
70m_k141_766853_len_2329-CJDMCFAD_181460_Heme_oxygenase_1_
EKGG159341_Heme_oxygenase_Prochlorococcus_sp_MIT_0603
EABX096461_Heme_oxygenase_Prochlorococcus_marinus_str_MIT_9211
EWP_0369007351_heme_oxygenase_Prochlorococcus_sp_MIT_0601
St16_S0090_3966_CAGHKFPH_25690_Heme_oxygenase_1_
EWP_0496915521_heme_oxygenase_Synechococcus_sp_WH_8103
EWP_0111270471_heme_oxygenase_Synechococcus_sp_WH_8102
St6_S0133_6712_OHKFMJEP_173416_Heme_oxygenase_1_
EWP_0097883611_heme_oxygenase_Synechococcus_sp_BL107
gene_327907_GeneMark_hmm_237_aa_53_766_175576_
gene_1105296_GeneMark_hmm_237_aa_2333_3046_581006_
EWP_0070996071_heme_oxygenase_Synechococcus_sp_RS9916
EWP_0385427951_heme_oxygenase_Synechococcus_sp_KORDI_100
EWP_0385516541_heme_oxygenase_Synechococcus_sp_KORDI_52
EWP_0113632011_heme_oxygenase_Synechococcus_sp_CC9605
EWP_0103142961_heme_oxygenase_Synechococcus_sp_CB0205
EWP_0103122651_heme_oxygenase_Synechococcus_sp_CB0101
EABM789761_Heme_oxygenase_Prochlorococcus_marinus_str_MIT_9303
ECAE218601_Heme_oxygenase_Prochlorococcus_marinus_str_MIT_9313
EKGG342831_Heme_oxygenase_Prochlorococcus_sp_MIT_0703
80m_St9_1238531-ANBNGGHB_182850_Heme_oxygenase_1_
120mF_k141_471082_len1942-OMEFNEKA_327588_Heme_oxygenase_1_
EWP_0480164491_heme_oxygenase_Synechococcus_sp_GFB01
EWP_0061725201_heme_oxygenase_Synechococcus_sp_WH_5701
EWP_0483477491_heme_oxygenase_Synechococcus_sp_WH_8020
EWP_0068537671_heme_oxygenase_Synechococcus_sp_WH_8016
gene_581610_GeneMark_hmm_212_aa_204_842_315852_
EWP_0119345731_heme_oxygenase_Synechococcus_sp_RCC307
EWP_0071009601_heme_oxygenase_Synechococcus_sp_RS9917
EKKZ112491_heme_oxygenase_Candidatus_Synechococcus_spongiarum_142
EYP_0020491241_Heme_oxygenase_chromatophore_Paulinella_chromatophora
VTLG01012686_HOT201112686_BCHIGMAC_02854_Heme_oxygenase
80m_St9_20397-ANBNGGHB_135738_hypothetical_protein_
St18_S0059_5137_BCDADANA_94882_Heme_oxygenase_2_
gene_1069770_GeneMark_hmm_218_aa_485_1141_528434_
MAVG-MED-2_KCKFIKKH_00276_Heme_oxygenase_1
St22_S0139_4459_OLDBHJIL_45901_hypothetical_protein_
Pelagiphage_HOT_VTLG01009199_FGLCGAHL_01545_hypothetical_protein_
ASF00634_putative_heme_oxygenase_uncultured_virus
Pelagiphage_PMP_MAVG_12_KCKFIKKH_05704_hypothetical_protein_
VTLG01003937_HOT20113937_BCHIGMAC_00597_Heme_oxygenase
QOI69018_putative_heme_oxygenase_protein_Pelagibacter_phage_Mosig_EXVC030M
EBAR366181_Heme_oxygenase_like_protein_uncultured_Mediterranean_phage_uvMED
EBAR362291_Heme_oxygenase_like_protein_uncultured_Mediterranean_phage_uvMED
Pelagiphage_PMP_MAVG_4_KCKFIKKH_02671_hypothetical_protein_
EWP_0065123541_heme_oxygenase_Synechococcus_sp_PCC_7305
EWP_0151937351_heme_oxygenase_Stanieria_cyanosphaera
EWP_0151895931_heme_oxygenase_Gloeocapsa_sp_PCC_7428
EWP_0027376751_heme_oxygenase_Microcystis_aeruginosa
St10_S0084_472_MPHBJNLH_80595_Heme_oxygenase_1_
St18_S0060_9576_BCDADANA_147842_Heme_oxygenase_1_
70m_k141_437116_len_1276-CJDMCFAD_48267_Heme_oxygenase_1_
70m_k141_670090_len_1602-CJDMCFAD_144383_Heme_oxygenase_1_
EAGN123091_heme_oxygenase_Prochlorococcus_phage_P_SSM5
St16_S0094_2164_CAGHKFPH_126578_Heme_oxygenase_1_
GP13_gt8_S0334_3223_ODNKDCGA_90518_Heme_oxygenase_1_
GP13_gt8_S0334_973_ODNKDCGA_77690_Heme_oxygenase_1_
01003955__HOT20113955_HFHAMEHD_129077_Heme_oxygenase_1_
90mk141_25091_len_1052-DAALLOGA_01395_Heme_oxygenase_1_
70m_k141_452890_len_1449-CJDMCFAD_54952_Heme_oxygenase_1_
01012790__HOT201112790_HFHAMEHD_417171_Heme_oxygenase_1_
01016178__HOT201116178_HFHAMEHD_526863_Heme_oxygenase_1_
St18_S0060_4350_BCDADANA_134375_Heme_oxygenase_1_
EAGH076071_heme_oxygenase_Cyanophage_S_SSM6a
EYP_0043243511_heme_oxygenase_Synechococcus_phage_S_SSM7
EADO983621_heme_oxygenase_Synechococcus_phage_S_SSM7
01014715__HOT201114715_HFHAMEHD_482990_Heme_oxygenase_1_
St20_S0075_30_NDKAGLHG_57253_Heme_oxygenase_1_
01011627__HOT201111627_HFHAMEHD_381280_Heme_oxygenase_1_
St10_S0084_405_MPHBJNLH_79931_Heme_oxygenase_1_
GP13_gt15_S0253_2169_JFGJECEK_17972_Heme_oxygenase_1_
EYP_0076738841_heme_oxygenase_Prochlorococcus_phage_MED4_213
EYP_0043226351_heme_oxygenase_Prochlorococcus_phage_P_HM1
EYP_0043235781_heme_oxygenase_Prochlorococcus_phage_P_HM2
St10_S0084_3587_MPHBJNLH_95850_Heme_oxygenase_1_
GP13_gt8_S0334_198_ODNKDCGA_69396_Heme_oxygenase_1_
01012254__HOT201112254_HFHAMEHD_396420_Heme_oxygenase_1_
01007538__HOT20117538_HFHAMEHD_247156_Heme_oxygenase_1_
EYP_0076750661_heme_oxygenase_Cyanophage_P_RSM6
St7_S0234_24_JMKBNAHG_97330_Heme_oxygenase_1_
St24_S0100_3053_JDOFPADL_44118_Heme_oxygenase_1_
GP13_gt3_S0432_3431_DCNCJNKA_59338_Heme_oxygenase_1_
GP13_gt15_S0253_2167_JFGJECEK_17952_Heme_oxygenase_1_
EABB258191_DNA_N1_methyladenine_dioxygenase_Synechococcus_sp_CC9902
EWP_0436917331_DNA_repair_protein_Synechococcus_sp_KORDI_49
EWP_0070981131_alkylated_DNA_repair_protein_Synechococcus_sp_RS9916
EWP_0068509071_alkylated_DNA_repair_protein_Synechococcus_sp_WH_8109
EWP_0103164841_alkylated_DNA_repair_protein_Synechococcus_sp_CB0205
EWP_0103066241_alkylated_DNA_repair_protein_Synechococcus_sp_CB0101
EWP_0251652931_DNA_repair_protein_Pseudomonas_taeanensis
EWP_0510154731_2OG_FeII_oxygenase_Alcanivorax_dieselolei

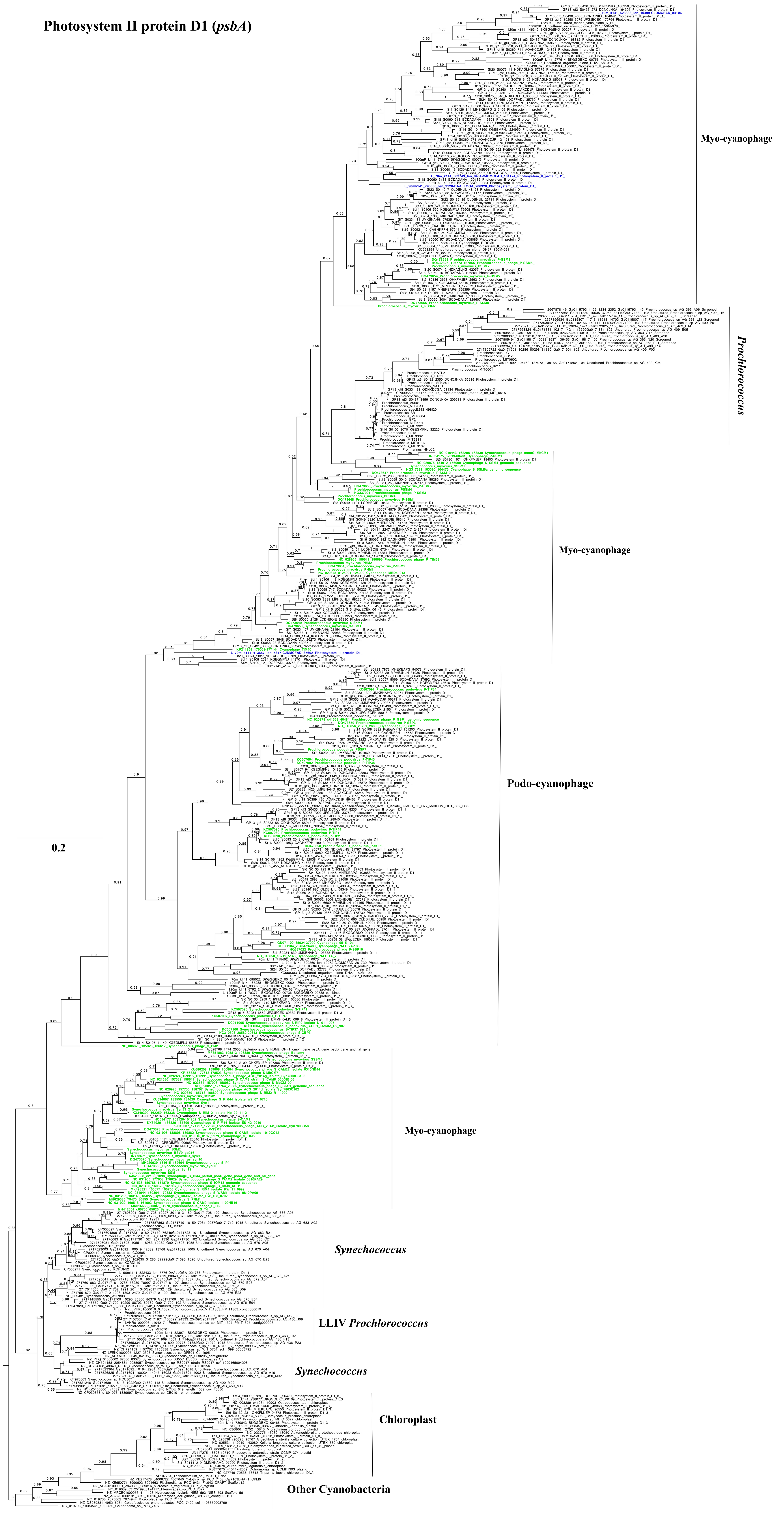

Photosystem II protein D1 (psbA)
Myo-cyanophage
Prochlorococcus
Myo-cyanophage
Podo-cyanophage
0.2
Myo-cyanophage
Synechococcus
LLIV Prochlorococcus
Synechococcus
Chloroplast
Other Cyanobacteria

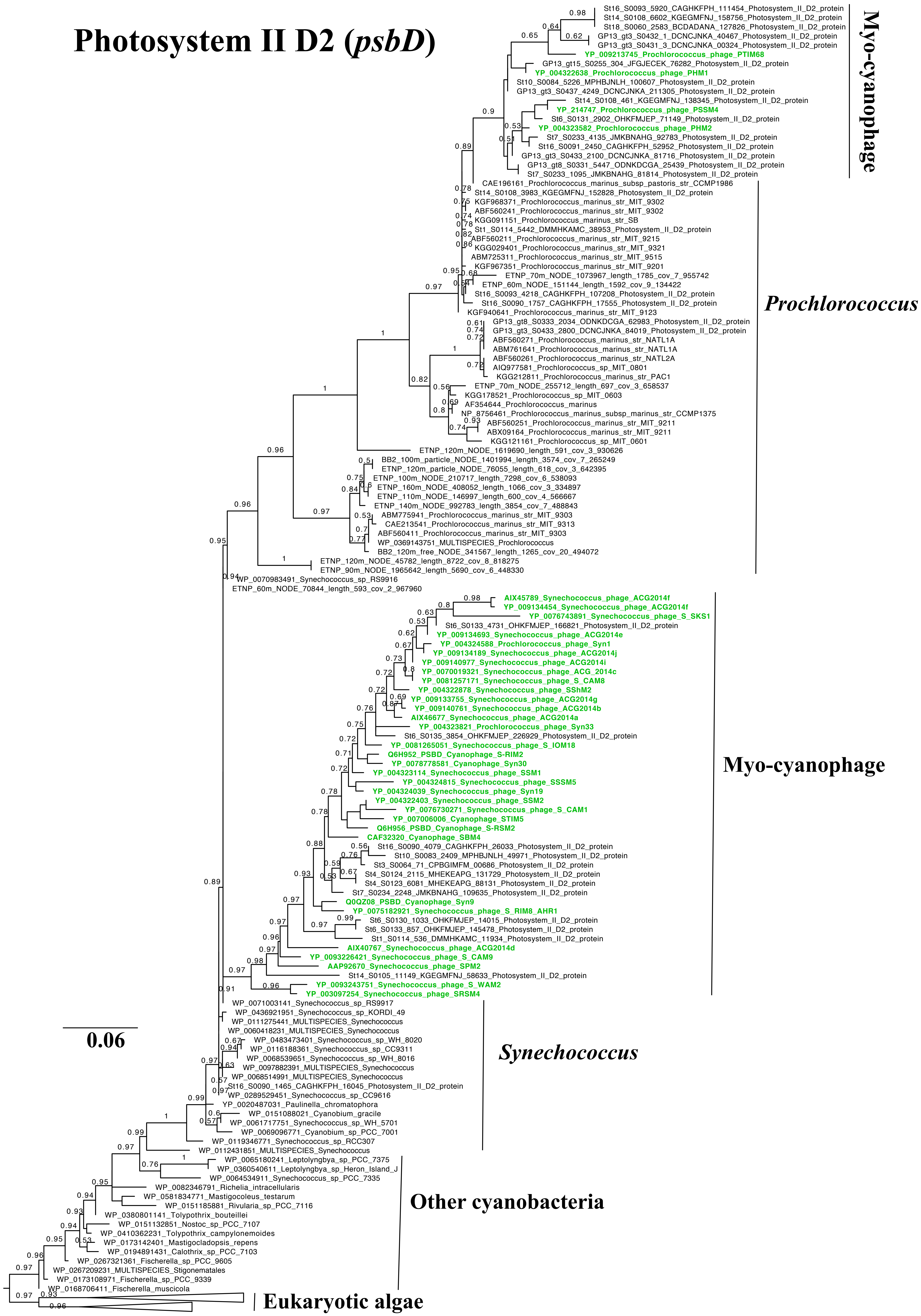
Photosystem II D2 (psbD)
Myo-cyanophage
Prochlorococcus
Myo-cyanophage
Synechococcus
Other cyanobacteria
Eukaryotic algae
0.06
St16_S0093_5920_CAGHKFPH_111454_Photosystem_II_D2_protein
St14_S0108_6602_KGEGMFNJ_158756_Photosystem_II_D2_protein
St18_S0060_2583_BCDADANA_127826_Photosystem_II_D2_protein
GP13_gt3_S0432_1_DCNCJNKA_40467_Photosystem_II_D2_protein
GP13_gt3_S0431_3_DCNCJNKA_00324_Photosystem_II_D2_protein
YP_009213745_Prochlorococcus_phage_PTIM68
GP13_gt15_S0255_304_JFGJECEK_76282_Photosystem_II_D2_protein
YP_004322638_Prochlorococcus_phage_PHM1
St10_S0084_5226_MPHBJNLH_100607_Photosystem_II_D2_protein
GP13_gt3_S0437_4249_DCNCJNKA_211305_Photosystem_II_D2_protein
St14_S0108_461_KGEGMFNJ_138345_Photosystem_II_D2_protein
YP_214747_Prochlorococcus_phage_PSSM4
St6_S0131_2902_OHKFMJEP_71149_Photosystem_II_D2_protein
YP_004323582_Prochlorococcus_phage_PHM2
St7_S0233_4135_JMKBNAHG_92783_Photosystem_II_D2_protein
St16_S0091_2450_CAGHKFPH_52952_Photosystem_II_D2_protein
GP13_gt3_S0433_2100_DCNCJNKA_81716_Photosystem_II_D2_protein
GP13_gt8_S0331_5447_ODNKDCGA_25439_Photosystem_II_D2_protein
St7_S0233_1095_JMKBNAHG_81814_Photosystem_II_D2_protein
CAE196161_Prochlorococcus_marinus_subsp_pastoris_str_CCMP1986
St14_S0108_3983_KGEGMFNJ_152828_Photosystem_II_D2_protein
KGF968371_Prochlorococcus_marinus_str_MIT_9302
ABF560241_Prochlorococcus_marinus_str_MIT_9302
KGG091151_Prochlorococcus_marinus_str_SB
St1_S0114_5442_DMMHKAMC_38953_Photosystem_II_D2_protein
ABF560211_Prochlorococcus_marinus_str_MIT_9215
KGG029401_Prochlorococcus_marinus_str_MIT_9321
ABM725311_Prochlorococcus_marinus_str_MIT_9515
KGF967351_Prochlorococcus_marinus_str_MIT_9201
ETNP_70m_NODE_1073967_length_1785_cov_7_955742
ETNP_60m_NODE_151144_length_1592_cov_9_134422
St16_S0093_4218_CAGHKFPH_107208_Photosystem_II_D2_protein
St16_S0090_1757_CAGHKFPH_17555_Photosystem_II_D2_protein
KGF940641_Prochlorococcus_marinus_str_MIT_9123
GP13_gt8_S0333_2034_ODNKDCGA_62983_Photosystem_II_D2_protein
GP13_gt3_S0433_2800_DCNCJNKA_84019_Photosystem_II_D2_protein
ABF560271_Prochlorococcus_marinus_str_NATL1A
ABM761641_Prochlorococcus_marinus_str_NATL1A
ABF560261_Prochlorococcus_marinus_str_NATL2A
AIQ977581_Prochlorococcus_sp_MIT_0801
KGG212811_Prochlorococcus_marinus_str_PAC1
ETNP_70m_NODE_255712_length_697_cov_3_658537
KGG178521_Prochlorococcus_sp_MIT_0603
AF354644_Prochlorococcus_marinus
NP_8756461_Prochlorococcus_marinus_subsp_marinus_str_CCMP1375
ABF560251_Prochlorococcus_marinus_str_MIT_9211
ABX09164_Prochlorococcus_marinus_str_MIT_9211
KGG121161_Prochlorococcus_sp_MIT_0601
ETNP_120m_NODE_1619690_length_591_cov_3_930626
BB2_100m_particle_NODE_1401994_length_3574_cov_7_265249
ETNP_120m_particle_NODE_76055_length_618_cov_3_642395
ETNP_100m_NODE_210717_length_7298_cov_6_538093
ETNP_160m_NODE_408052_length_1066_cov_3_334897
ETNP_110m_NODE_146997_length_600_cov_4_566667
ETNP_140m_NODE_992783_length_3854_cov_7_488843
ABM775941_Prochlorococcus_marinus_str_MIT_9303
CAE213541_Prochlorococcus_marinus_str_MIT_9313
ABF560411_Prochlorococcus_marinus_str_MIT_9303
WP_0369143751_MULTISPECIES_Prochlorococcus
BB2_120m_free_NODE_341567_length_1265_cov_20_494072
ETNP_120m_NODE_45782_length_8722_cov_8_818275
ETNP_90m_NODE_1965642_length_5690_cov_6_448330
WP_0070983491_Synechococcus_sp_RS9916
ETNP_60m_NODE_70844_length_593_cov_2_967960
AIX45789_Synechococcus_phage_ACG2014f
YP_009134454_Synechococcus_phage_ACG2014f
YP_0076743891_Synechococcus_phage_S_SKS1
St6_S0133_4731_OHKFMJEP_166821_Photosystem_II_D2_protein
YP_009134693_Synechococcus_phage_ACG2014e
YP_004324588_Prochlorococcus_phage_Syn1
YP_009134189_Synechococcus_phage_ACG2014j
YP_009140977_Synechococcus_phage_ACG2014i
YP_0070019321_Synechococcus_phage_ACG_2014c
YP_0081257171_Synechococcus_phage_S_CAM8
YP_004322878_Synechococcus_phage_SShM2
YP_009133755_Synechococcus_phage_ACG2014g
YP_009140761_Synechococcus_phage_ACG2014b
AIX46677_Synechococcus_phage_ACG2014a
YP_004323821_Prochlorococcus_phage_Syn33
St6_S0135_3854_OHKFMJEP_226929_Photosystem_II_D2_protein
YP_0081265051_Synechococcus_phage_S_IOM18
Q6H952_PSBD_Cyanophage_S-RIM2
YP_0078778581_Cyanophage_Syn30
YP_004323114_Synechococcus_phage_SSM1
YP_004324815_Synechococcus_phage_SSSM5
YP_004324039_Synechococcus_phage_Syn19
YP_004322403_Synechococcus_phage_SSM2
YP_0076730271_Synechococcus_phage_S_CAM1
YP_007006006_Cyanophage_STIM5
Q6H956_PSBD_Cyanophage_S-RSM2
CAF32320_Cyanophage_SBM4
St16_S0090_4079_CAGHKFPH_26033_Photosystem_II_D2_protein
St10_S0083_2409_MPHBJNLH_49971_Photosystem_II_D2_protein
St3_S0064_71_CPBGIMFM_00686_Photosystem_II_D2_protein
St4_S0124_2115_MHEKEAPG_131729_Photosystem_II_D2_protein
St4_S0123_6081_MHEKEAPG_88131_Photosystem_II_D2_protein
St7_S0234_2248_JMKBNAHG_109635_Photosystem_II_D2_protein
Q0QZ08_PSBD_Cyanophage_Syn9
YP_0075182921_Synechococcus_phage_S_RIM8_AHR1
St6_S0130_1033_OHKFMJEP_14015_Photosystem_II_D2_protein
St6_S0133_857_OHKFMJEP_145478_Photosystem_II_D2_protein
St1_S0114_536_DMMHKAMC_11934_Photosystem_II_D2_protein
AIX40767_Synechococcus_phage_ACG2014d
YP_0093226421_Synechococcus_phage_S_CAM9
AAP92670_Synechococcus_phage_SPM2
St14_S0105_11149_KGEGMFNJ_58633_Photosystem_II_D2_protein
YP_0093243751_Synechococcus_phage_S_WAM2
YP_003097254_Synechococcus_phage_SRSM4
WP_0071003141_Synechococcus_sp_RS9917
WP_0436921951_Synechococcus_sp_KORDI_49
WP_0111275441_MULTISPECIES_Synechococcus
WP_0060418231_MULTISPECIES_Synechococcus
WP_0483473401_Synechococcus_sp_WH_8020
WP_0116188361_Synechococcus_sp_CC9311
WP_0068539651_Synechococcus_sp_WH_8016
WP_0097882391_MULTISPECIES_Synechococcus
WP_0068514991_MULTISPECIES_Synechococcus
St16_S0090_1465_CAGHKFPH_16045_Photosystem_II_D2_protein
WP_0289529451_Synechococcus_sp_CC9616
YP_0020487031_Paulinella_chromatophora
WP_0151088021_Cyanobium_gracile
WP_0061717751_Synechococcus_sp_WH_5701
WP_0069096771_Cyanobium_sp_PCC_7001
WP_0119346771_Synechococcus_sp_RCC307
WP_0112431851_MULTISPECIES_Synechococcus
WP_0065180241_Leptolyngbya_sp_PCC_7375
WP_0360540611_Leptolyngbya_sp_Heron_Island_J
WP_0064534911_Synechococcus_sp_PCC_7335
WP_0082346791_Richelia_intracellularis
WP_0581834771_Mastigocoleus_testarum
WP_0151185881_Rivularia_sp_PCC_7116
WP_0380801141_Tolypothrix_bouteillei
WP_0151132851_Nostoc_sp_PCC_7107
WP_0410362231_Tolypothrix_campylonemoides
WP_0173142401_Mastigocladopsis_repens
WP_0194891431_Calothrix_sp_PCC_7103
WP_0267321361_Fischerella_sp_PCC_9605
WP_0267209231_MULTISPECIES_Stigonematales
WP_0173108971_Fischerella_sp_PCC_9339
WP_0168706411_Fischerella_muscicola

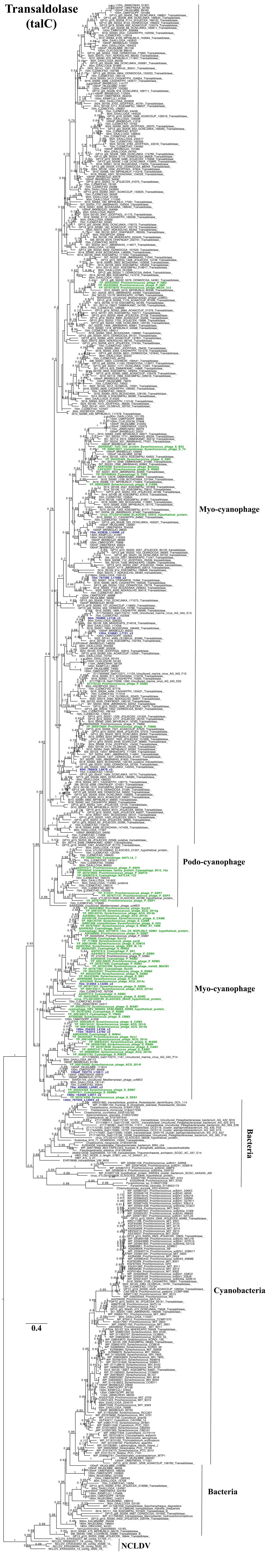
Transaldolase
(talC)
Myo-cyanophage
Podo-cyanophage
Myo-cyanophage
Bacteria
Cyanobacteria
Bacteria
NCLDV
0.4

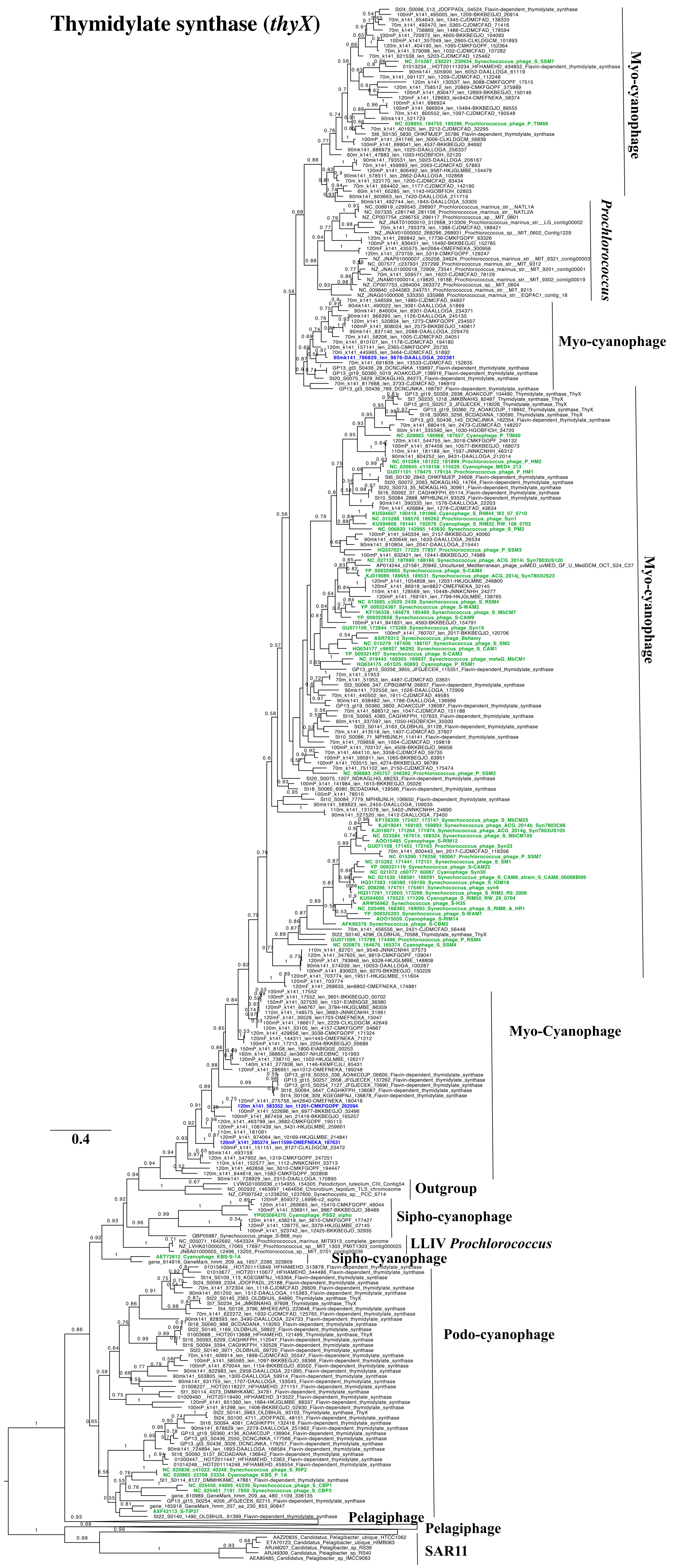

Thymidylate synthase (thyX)
Myo-cyanophage
Prochlorococcus
Myo-cyanophage
Myo-cyanophage
Myo-Cyanophage
0.4
Outgroup
Sipho-cyanophage
LLIV Prochlorococcus
Sipho-cyanophage
Podo-cyanophage
Pelagiphage
Pelagiphage
SAR11

Orotate phosphoribosyltransferase (*pyrE*)

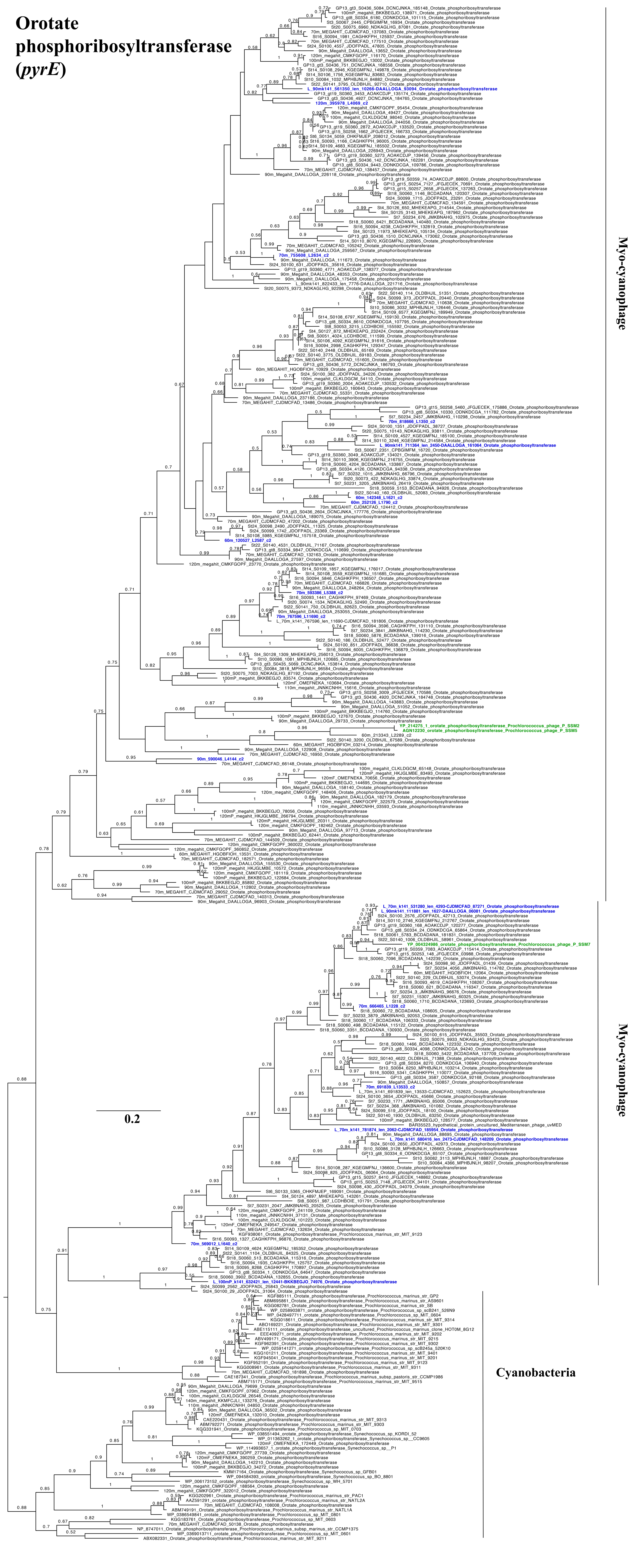

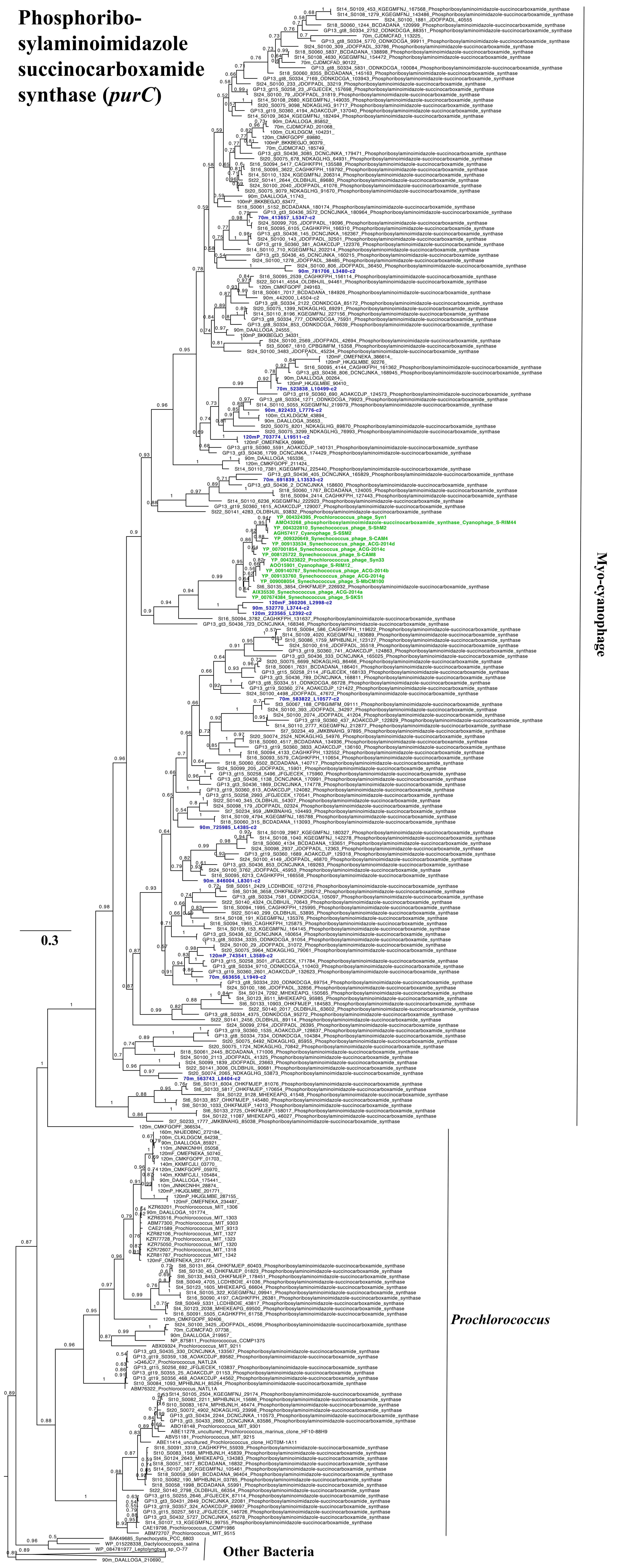
Phosphoribo-
sylaminoimidazole
succinocarboxamide
synthase (purC)
Myo-cyanophage
Prochlorococcus
Other Bacteria
0.3

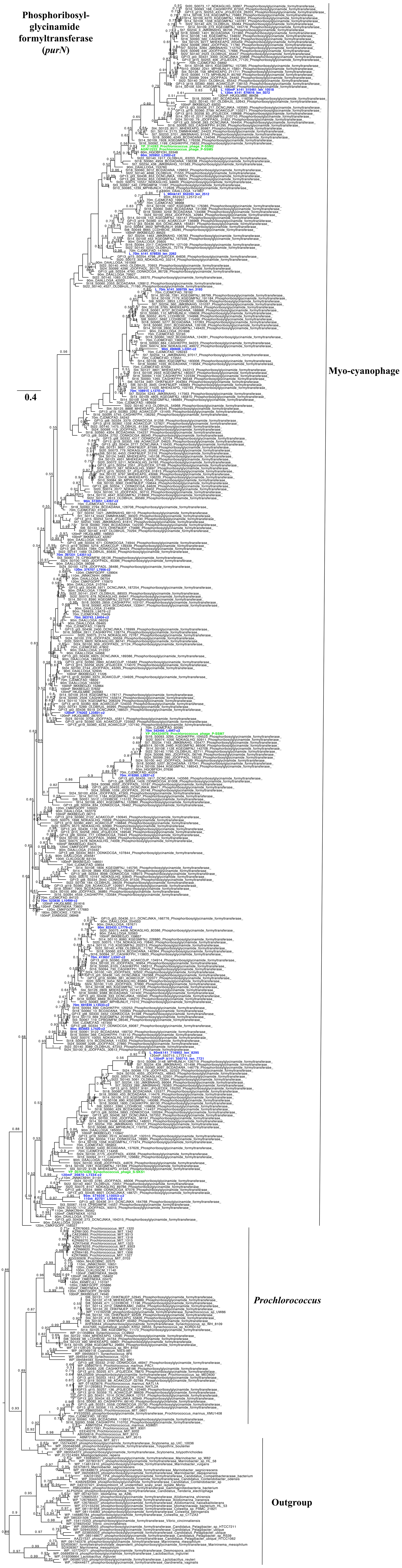

Phosphoribosyl-glycinamide formyltransferase (purN)
0.4
Myo-cyanophage
Prochlorococcus
Outgroup

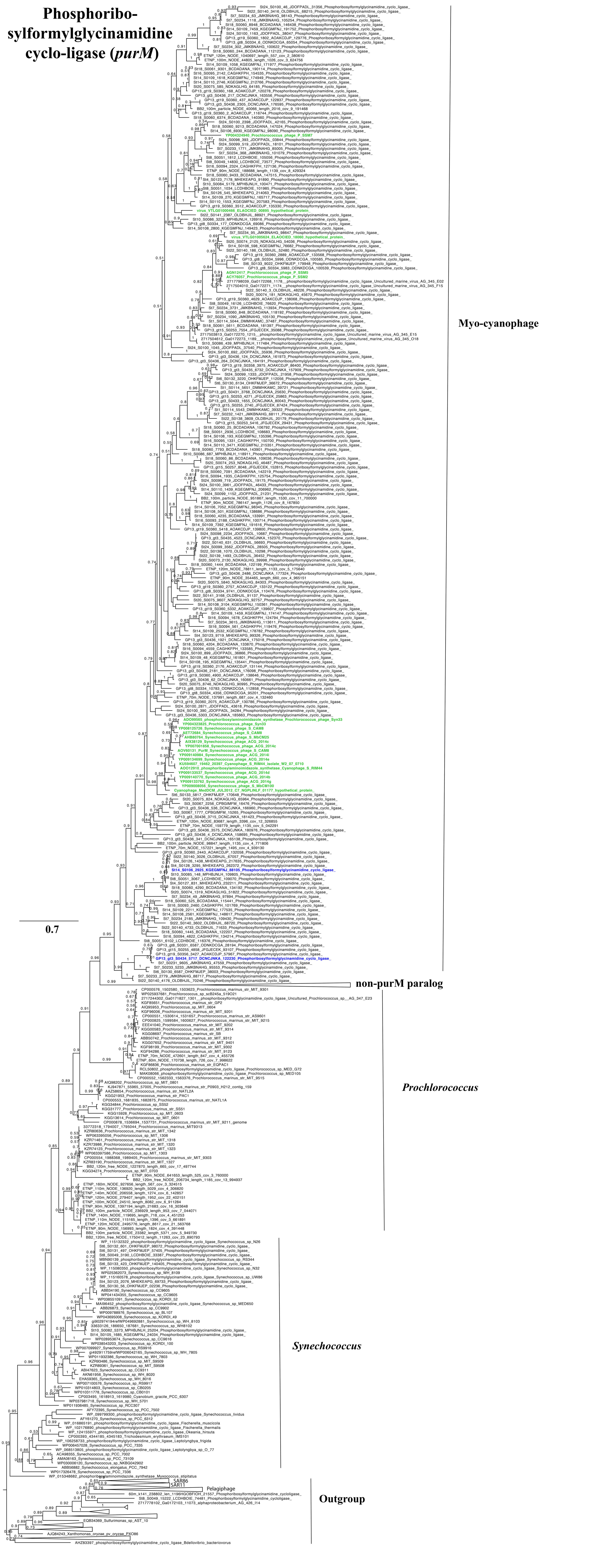

Phosphoribo-
sylformylglycinamidine
cyclo-ligase (*purM*)
Myo-cyanophage
0.7
non-purM paralog
Prochlorococcus
Synechococcus
Outgroup
Pelagiphage
SAR86
SAR11

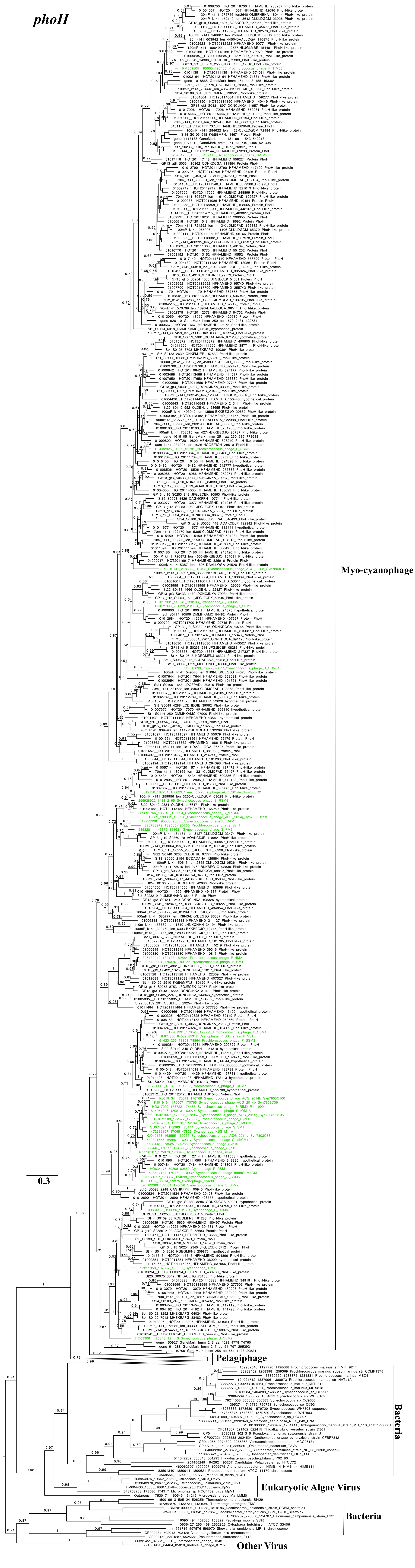
phoH
Myo-cyanophage
Pelagiphage
Bacteria
Eukaryotic Algae Virus
Bacteria
Other Virus
0.3
